# Supplementary material for: Revisiting functioning recovery in persons with spinal cord injury undergoing first rehabilitation: Trajectory and network analysis of a Swiss cohort study
Source: PLoS One. 2024 Feb 9;19(2):e0297682. doi: 10.1371/journal.pone.0297682 (PMC10857630; doi:10.1371/journal.pone.0297682)
Supplement: S1 Table — Variables selected for the network analysis are marked in green. (PDF) [file pone.0297682.s001.pdf]

S1 Table. Overview about the selection of relevant functioning problems and corresponding SwiSCI Inception Cohort variables according to Brief ICF Core Set for SCI (early post-acute situation)[1] and selected categories according to Ballert et al[2]. Variables selected for the network analysis are marked in green.

ICF Code	SwiSCI dataset		Missing observations T1, N (%)		Missing observations T4, N (%)		Inclusion decision (Yes/No)	Comment
	Instrument	Name of candidate SwiSCI variable	Stable high functioning class (N=239)	Moderate functioning improvement class (N=753)	Stable high functioning class (N=239)	Moderate functioning improvement class (N=753)		
				Brief ICF Core Set for SCI				
b152 Emotional functions	SF-36	vitality_nervous					No	No T4 assessment
		vitality_down					No	No T4 assessment
		vitality_calm					No	No T4 assessment
		vitality_depressed					No	No T4 assessment
		vitality_happy					No	No T4 assessment
	HADS	mood_enjoy	104 (43.51)	374 (49.67)	101 (42.26)	374 (49.67)	No	Missingness
		mood_laugh	104 (43.51)	369 (49.00)	100 (41.84)	371 (49.27)	No	Missingness
		mood_cheerful	105 (43.93)	370 (49.14)	102 (42.68)	371 (49.27)	No	Missingness
		mood_appearance	104 (43.51)	371 (49.27)	101 (42.26)	372 (49.40)	No	Missingness
		mood_enjoyment	106 (44.35)	374 (49.67)	101 (42.26)	373 (49.54)	No	Missingness
		mood_book	104 (43.51)	374 (49.67)	101 (42.26)	374 (49.67)	No	Missingness
		mood_stressed	105 (43.93)	369 (49.00)	100 (41.84)	371 (49.27)	No	Missingness
		mood_scared	105 (43.93)	372 (49.40)	101 (42.26)	372 (49.40)	No	Missingness
		mood_worry	104 (43.51)	370 (49.14)	101 (42.26)	371 (49.27)	No	Missingness
		mood_relax	104 (43.51)	369 (49.00)	101 (42.26)	371 (49.27)	No	Missingness
		mood_fear	104 (43.51)	371 (49.27)	101 (42.26)	372 (49.40)	No	Missingness
		mood_restless	105 (43.93)	371 (49.27)	101 (42.26)	371 (49.27)	No	Missingness
		mood_panic	104 (43.51)	373 (49.54)	101 (42.26)	374 (49.67)	No	Missingness
	SHS	*drugs*					No	No T4 assessment
		medicine					No	No T4 assessment
	IES-6	pts_agitated					No	No T4 assessment
		pts_alert					No	No T4 assessment
b280 Sensation of pain	ISCoS Pain basic data set	pain	119 (49.79)	451 (59.89)	103 (43.10)	374 (49.67)	No	Missingness
	BPI	*painzone_1**	237 (99.16)	751 (99.73)	236 (98.74)	750 (99.60)	No	Missingness
		*painzone_50**	233 (97.49)	746 (99.07)	233 (97.49)	747 (99.20)	No	Missingness

[1] Kirchberger I, Cieza A, Biering-Sørensen F, Baumberger M, Charlifue S, Post MW et al. ICF Core Sets for individuals with spinal cord injury in the early post-acute context. Spinal Cord 2010;48(4):297-304.
[2] Ballert C, Oberhauser C, Biering-Sørensen F, Stucki G, Cieza A. Explanatory power does not equal clinical importance: study of the use of the Brief ICF Core Sets for Spinal Cord Injury with a purely statistical approach. Spinal Cord 2012;50(10):734-9.

S1 Table. Overview about the selection of relevant functioning problems and corresponding SwiSCI Inception Cohort variables according to Brief ICF Core Set for SCI (early post-acute situation)[1] and selected categories according to Ballert et al[2]. Variables selected for the network analysis are marked in green.

ICF Code	SwiSCI dataset		Missing observations T1, N (%)		Missing observations T4, N (%)		Inclusion decision (Yes/No)	Comment
	Instrument	Name of candidate SwiSCI variable	Stable high functioning class (N=239)	Moderate functioning improvement class (N=753)	Stable high functioning class (N=239)	Moderate functioning improvement class (N=753)		
	SCIPI	*pain_intensity_max*	181 (75.73)	596 (79.15)	177 (74.06)	558 (74.10)	No	Missingness
		pain_intensity_avg	177 (74.06)	593 (78.75)	179 (74.90)	558 (74.10)	No	Missingness
		pain_elec	218 (91.21)	690 (91.63)	210 (87.87)	656 (87.12)	No	Missingness
		pain_prick	218 (91.21)	688 (91.37)	211 (88.28)	656 (87.12)	No	Missingness
		pain_deep	219 (91.63)	690 (91.63)	212 (88.70)	656 (87.12)	No	Missingness
		pain_numb	218 (91.21)	689 (91.50)	210 (87.87)	656 (87.12)	No	Missingness
	SCI-SCS	problem_pain					No	No T1/T4 assessment
b440 Respiration functions	SCIM III	scim_respiration	15 (6.28)	32 (4.25)	4 (1.67)	8 (1.06)	Yes	
	ISCoS pulmonary function data set	*pulmonary*	3 (1.26)	5 (0.66)	73 (30.54)	290 (38.51)	No	Missingness
		spiro_fvc	137 (57.32)	466 (61.89)	96 (40.17)	371 (49.27)	No	Missingness
		spiro_fev1	137 (57.32)	466 (61.89)	96 (40.17)	371 (49.27)	No	Missingness
		spiro_pef	137 (57.32)	466 (61.89)	96 (40.17)	371 (49.27)	No	Missingness
		spiro_pef_unit	139 (58.16)	473 (62.82)	98 (41.00)	386 (51.26)	No	Missingness
	SCI-SCS	problem_respiratory					No	No T1/T4 assessment
	SHS	problem_asthma					No	No T1/T4 assessment
	NA	problem_copd					No	No T1/T4 assessment
	NA	problem_pneumonia					No	No T1/T4 assessment
b525 Defecation functions	ISCoS Core Data Set / ISNCSCI	fneurol_contraction					No	No T4 assessment
		fneurol_sensation					No	No T4 assessment
	ISCoS bowel function basic data set	bowel_defecate	94 (39.33)	354 (47.01)	82 (34.31)	314 (41.70)	No	Missingness
		bowel_care	92 (38.49)	333 (44.22)	80 (33.47)	312 (41.43)	No	Missingness
		bowel_defecation_tm	132 (55.23)	414 (54.98)	118 (49.37)	353 (46.88)	No	Missingness
		bowel_defecation_freq	103 (43.10)	349 (46.35)	90 (37.66)	314 (41.70)	No	Missingness
		bowel_fecal_freq	112 (46.86)	414 (54.98)	94 (39.33)	365 (48.47)	No	Missingness
		bowel_perianal					No	Not available in dataset**
		bowel_perianal_1					No	Sub question
	SCIM III	*scim_bowel*	15 (6.28)	32 (4.25)	4 (1.67)	8 (1.06)	Yes	
		neurol_contraction					No	Neurologic examination

S1 Table. Overview about the selection of relevant functioning problems and corresponding SwiSCI Inception Cohort variables according to Brief ICF Core Set for SCI (early post-acute situation)[1] and selected categories according to Ballert et al[2]. Variables selected for the network analysis are marked in green.

ICF Code	SwiSCI dataset		Missing observations T1, N (%)		Missing observations T4, N (%)		Inclusion decision (Yes/No)	Comment
	Instrument	Name of candidate SwiSCI variable	Stable high functioning class (N=239)	Moderate functioning improvement class (N=753)	Stable high functioning class (N=239)	Moderate functioning improvement class (N=753)		
	ISCoS Core Data Set / ISNCSCI	neurol_sensation					No	Neurologic examination
	SCI-SCS	problem_bowel					No	No T1/T4 assessment
b620 Urination functions	SCIM III	*scim_bladder*	15 (6.28)	32 (4.25)	4 (1.67)	9 (1.20)	Yes	
	ISCoS Lower urinary tract function basic data set	urin_empty	94 (39.33)	323 (42.90)	78 (32.64)	303 (40.24)	No	Missingness
		urin_empty_1					No	Sub question
		urin_empty_2					No	Sub question
		urin_empty_3					No	Sub question
		urin_empty_4					No	Sub question
		urin_empty_5					No	Sub question
		urin_empty_6					No	Sub question
		urin_empty_7					No	Sub question
		urin_empty_8					No	Sub question
		urin_empty_9					No	Sub question
		urin_empty_10					No	Sub question
		urin_empty_num	108 (45.19)	388 (51.53)	101 (42.26)	357 (47.41)	No	Missingness
		urin_incontinence	109 (45.61)	452 (60.03)	92 (38.49)	381 (50.60)	No	Missingness
		urin_appliance					No	Not available in dataset**
		urin_drugs					No	Not available in dataset**
	SCI-SCS	problem_bladder					No	No T1/T4 assessment
		problem_urinary					No	No T1/T4 assessment
b730 Muscle power functions	ISCoS Core Data Set / ISNCSCI	fneurol_contraction					No	No T4 assessment
		fneurol_motor_all					No	No T4 assessment
		fneurol_ais					No	No T4 assessment
		neurol_contraction					No	Neurologic examination
		neurol_motor_all					No	Neurologic examination
		neurol_ais					No	Neurologic examination

S1 Table. Overview about the selection of relevant functioning problems and corresponding SwiSCI Inception Cohort variables according to Brief ICF Core Set for SCI (early post-acute situation)[1] and selected categories according to Ballert et al[2]. Variables selected for the network analysis are marked in green.

ICF Code	SwiSCI dataset		Missing observations T1, N (%)		Missing observations T4, N (%)		Inclusion decision (Yes/No)	Comment
	Instrument	Name of candidate SwiSCI variable	Stable high functioning class (N=239)	Moderate functioning improvement class (N=753)	Stable high functioning class (N=239)	Moderate functioning improvement class (N=753)		
	Hand Dynamo-meter	ah_grip_r_2_m1					No	Summarized by ah_grip_r_2_mean
		ah_grip_r_2_m2					No	
		ah_grip_r_2_m3					No	
		ah_grip_r_2_mean	150 (62.76)	511 (67.86)	124 (51.88)	431 (57.24)	No	
		ah_grip_l_2_m1					No	Summarized by ah_grip_l_2_mean
		ah_grip_l_2_m2					No	
		ah_grip_l_2_m3					No	
		ah_grip_l_2_mean	150 (62.76)	512 (67.99)	125 (52.30)	431 (57.24)	No	
b735 Muscle tone functions	Muscle Tone Scale	muscle_test					No	No T1/T4 assessment
	MAS	spasticity_biceps_r	117 (48.95)	447 (59.36)	95 (39.75)	341 (45.29)	No	Missingness
		spasticity_biceps_l	117 (48.95)	448 (59.50)	95 (39.75)	340 (45.15)	No	Missingness
		spasticity_gastrocnemius_r	117 (48.95)	450 (59.76)	96 (40.17)	342 (45.42)	No	Missingness
		spasticity_gastrocnemius_l	117 (48.95)	451 (59.89)	95 (39.75)	343 (45.55)	No	Missingness
	SCI-SCS	problem_spasticity					No	No T1/T4 assessment
b810 Protective functions of the skin	ISCoS Skin and thermo-regulation function basic data set	decubitus	73 (30.54)	228 (30.28)	58 (24.27)	217 (28.82)	No	Missingness
	SCI-SCS	problem_pressure					No	No T1/T4 assessment
d410 Changing basic body position	SCIM III	scim_mobility_bed	15 (6.28)	32 (4.25)	4 (1.67)	8 (1.06)	Yes	
		scim_bed_wheelchair	14 (5.86)	33 (4.38)	4 (1.67)	12 (1.59)	Yes	
	SCIM-SR	scim_add_1					No	No T1/T4 assessment
d420 Transferring oneself	SCIM III	scim_bed_wheelchair					Yes	See d410 Changing basic body position
		scim_wheelchair_toilet	23 (9.62)	63 (8.37)	4 (1.67)	12 (1.59)	Yes	
		scim_wheelchair_car	14 (5.86)	32 (4.25)	5 (2.09)	11 (1.46)	Yes	
		scim_ground_wheelchair	15 (6.28)	32 (4.25)	4 (1.67)	14 (1.86)	Yes	
	SCI-SCS	problem_hypotension					No	No T1/T4 assessment
d445 Hand and arm use	Hand Dynamo-meter	*ah_grip_r_2_m1*					No	See b730 Muscle power functions
		ah_grip_r_2_m2					No	

S1 Table. Overview about the selection of relevant functioning problems and corresponding SwiSCI Inception Cohort variables according to Brief ICF Core Set for SCI (early post-acute situation)[1] and selected categories according to Ballert et al[2]. Variables selected for the network analysis are marked in green.

	SwiSCI dataset		Missing observations T1, N (%)		Missing observations T4, N (%)			
ICF Code	Instrument	Name of candidate SwiSCI variable	Stable high functioning class (N=239)	Moderate functioning improvement class (N=753)	Stable high functioning class (N=239)	Moderate functioning improvement class (N=753)	Inclusion decision (Yes/No)	Comment
		ah_grip_r_2_m3					No	
		ah_grip_r_2_mean					No	
		ah_grip_l_2_m1					No	
		ah_grip_l_2_m2					No	
		ah_grip_l_2_m3					No	
		ah_grip_l_2_mean					No	
	NA	*problem_sleep*					No	No T1/T4 assessment
d450 Walking	SCIM III	scim_indoor	15 (6.28)	32 (4.25)	4 (1.67)	14 (1.86)	Yes	
		scim_moderate	15 (6.28)	32 (4.25)	4 (1.67)	14 (1.86)	Yes	
		scim_outdoor	15 (6.28)	32 (4.25)	4 (1.67)	14 (1.86)	Yes	
d510 Washing oneself	SCIM III	scim_bathing_upper	15 (6.28)	32 (4.25)	4 (1.67)	8 (1.06)	Yes	
		scim_bathing_lower	15 (6.28)	32 (4.25)	4 (1.67)	8 (1.06)	Yes	
		scim_grooming	15 (6.28)	32 (4.25)	4 (1.67)	8 (1.06)	Yes	
d530 Toileting	ISCoS bowel function basic data set	bowel_care_1					No	Sub question
		bowel_care_2					No	Sub question
		bowel_care_3					No	Sub question
		bowel_care_4					No	Sub question
		bowel_care_5					No	Sub question
		bowel_care_6					No	Sub question
		bowel_care_7					No	Sub question
		bowel_care_8					No	Sub question
		bowel_care_9					No	Sub question
	SCIM III	scim_bladder	15 (6.28)	32 (4.25)	4 (1.67)	9 (1.20)	Yes	
		scim_bowel	15 (6.28)	32 (4.25)	4 (1.67)	8 (1.06)	Yes	
		scim_toilet	15 (6.28)	32 (4.25)	4 (1.67)	8 (1.06)	Yes	
		scim_wheelchair_toilet	23 (9.62)	63 (8.37)	4 (1.67)	12 (1.59)	Yes	
d540 Dressing	SCIM III	scim_dressing_upper	15 (6.28)	32 (4.25)	4 (1.67)	8 (1.06)	Yes	
		scim_dressing_lower	15 (6.28)	32 (4.25)	4 (1.67)	8 (1.06)	Yes	
d550 Eating	SCIM III	scim_feeding	15 (6.28)	32 (4.25)	4 (1.67)	8 (1.06)	Yes	
d560 Drinking	SCIM III	scim_feeding					Yes	See d550 Eating

S1 Table. Overview about the selection of relevant functioning problems and corresponding SwiSCI Inception Cohort variables according to Brief ICF Core Set for SCI (early post-acute situation)[1] and selected categories according to Ballert et al[2]. Variables selected for the network analysis are marked in green.

ICF Code	SwiSCI dataset		Missing observations T1, N (%)		Missing observations T4, N (%)		Inclusion decision (Yes/No)	Comment
	Instrument	Name of candidate SwiSCI variable	Stable high functioning class (N=239)	Moderate functioning improvement class (N=753)	Stable high functioning class (N=239)	Moderate functioning improvement class (N=753)		
				Statistical Set				
b126 Tempera-ment and personality functions	GSES	*skills_cope*	108 (45.19)	385 (51.13)	106 (44.35)	386 (51.26)	No	Missingness
	RSES	*belief_qualities*					No	No T4 assessment
		belief_useless					No	No T4 assessment
		belief_useful					No	No T4 assessment
		belief_positive					No	No T4 assessment
		belief_contented					No	No T1 assessment
		belief_be_good					No	No T1 assessment
		belief_qualities_trait					No	No T1 assessment
		belief_others					No	No T1 assessment
		belief_proud					No	No T1 assessment
		belief_useless_trait					No	No T1 assessment
		belief_useful_trait					No	No T1 assessment
		belief_respect					No	No T1 assessment
		belief_fail					No	No T1 assessment
		belief_positive_trait					No	No T1 assessment
	LOT	*optimism_expectation*	109 (45.61)	395 (52.46)	102 (42.68)	388 (51.53)	No	Missingness
		optimism_go_wrong	113 (47.28)	406 (53.92)	104 (43.51)	389 (51.66)	No	Missingness
		optimism_optimistic	110 (46.03)	394 (52.32)	103 (43.10)	387 (51.39)	No	Missingness
		optimism_develop	109 (45.61)	399 (52.99)	102 (42.68)	388 (51.53)	No	Missingness
		optimism_no_rely	109 (45.61)	399 (52.99)	102 (42.68)	388 (51.53)	No	Missingness
		optimism_good_things	111 (46.44)	399 (52.99)	103 (43.10)	391 (51.93)	No	Missingness
b130 Energy and drive functions	SF-36	vitality_drive					No	No T4 assessment
		vitality_energetic					No	No T4 assessment
		vitality_exhausted					No	No T4 assessment
		vitality_tired					No	No T4 assessment
	HADS	mood_slowed	104 (43.51)	372 (49.40)	101 (42.26)	371 (49.27)	No	Missingness
	SHP	*optimism_energy*	111 (46.44)	389 (51.66)	102 (42.68)	386 (51.26)	No	Missingness
b270 Sensory functions related to	ISCoS Core Data Set / ISNCSCI	fneurol_sensory_all					No	No T4 assessment
		fneurol_ais					No	No T4 assessment

S1 Table. Overview about the selection of relevant functioning problems and corresponding SwiSCI Inception Cohort variables according to Brief ICF Core Set for SCI (early post-acute situation)[1] and selected categories according to Ballert et al[2]. Variables selected for the network analysis are marked in green.

ICF Code	SwiSCI dataset		Missing observations T1, N (%)		Missing observations T4, N (%)		Inclusion decision (Yes/No)	Comment
	Instrument	Name of candidate SwiSCI variable	Stable high functioning class (N=239)	Moderate functioning improvement class (N=753)	Stable high functioning class (N=239)	Moderate functioning improvement class (N=753)		
temperature and other stimuli		neurol_sensory_all					No	Neurologic examination
		neurol_ais					No	Neurologic examination
	SCI-SCS	problem_injury					No	No T1/T4 assessment
b415 Blood vessel functions	CIRS	comorb_vascular_1					No	No T4 assessment
	ISCoS cardiovascular function data set	cardiac_function_6					No	Sub question
		cardiac_function_10					No	Sub question
	ISCoS bowel function basic data set	bowel_perianal_1					No	Sub question
	SHS	problem_stroke					No	No T1/T4 assessment
	NA	problem_thromb_leg					No	No T1/T4 assessment
	NA	problem_thromb_lung					No	No T1/T4 assessment
	SCI-SCS	problem_vascular					No	No T1/T4 assessment
b430 Haematolo-gical system functions	CIRS	comorb_hematopoietic_1					No	No T1/T4 assessment
		shc_hematopoietic_1					No	Not available in dataset**
b445 Respiratory muscle functions	ISCoS pulmonary function data set	pulmonary					No	See b440 Respiration function
b550 Thermo-regulatory functions								No variables identified
b670 Sensations associated with genital and reproductive functions								No variables identified
b710 Mobility of joint functions	NA	rom_shoulder_r_abduction*	111 (46.44)	399 (52.99)	103 (43.10)	357 (47.41)	No	Missingness
		rom_elbow_r_flexion*	113 (47.28)	399 (52.99)	103 (43.10)	357 (47.41)	No	Missingness
		rom_wrist_r_flexion*	116 (48.54)	403 (53.52)	105 (43.93)	360 (47.81)	No	Missingness
		rom_hip_r_flexion*	110 (46.03)	365 (48.47)	99 (41.42)	335 (44.49)	No	Missingness

S1 Table. Overview about the selection of relevant functioning problems and corresponding SwiSCI Inception Cohort variables according to Brief ICF Core Set for SCI (early post-acute situation)[1] and selected categories according to Ballert et al[2]. Variables selected for the network analysis are marked in green.

	SwiSCI dataset		Missing observations T1, N (%)		Missing observations T4, N (%)			
ICF Code	Instrument	Name of candidate SwiSCI variable	Stable high functioning class (N=239)	Moderate functioning improvement class (N=753)	Stable high functioning class (N=239)	Moderate functioning improvement class (N=753)	Inclusion decision (Yes/No)	Comment
		rom_knee_r_flexion*	111 (46.44)	365 (48.47)	99 (41.42)	335 (44.49)	No	Missingness
		rom_ankle_r_deextension*	111 (46.44)	367 (48.74)	99 (41.42)	336 (44.62)	No	Missingness
		problem_contractures					No	No T1/T4 assessment
		problem_arthritis					No	No T1/T4 assessment
		problem_arthrosis					No	No T1/T4 assessment
b715 Stability of joint functions								No variables identified
b755 Involuntary movement reaction functions								No variables identified
b760 Control of voluntary movement functions								No variables identified
b780 Sensations related to muscle and movement functions								No variables identified
d240 Handling stress and other psycho-logical demands	IMPACT-S	activity_planning_limit					No	No T1/T4 assessment
	SF-36***	*Mental health subscale*					No	No T4 assessment
	Distress thermo-meter	*strain*	118 (49.37)	452 (60.03)	101 (42.26)	378 (50.20)	No	Missingness
	Brief COPE***						No	No T1/T4 assessment
d465 Moving around using equipment	SCIM III	scim_indoor					Yes	See d450 Walking
		scim_moderate					Yes	See d450 Walking
		scim_outdoor					Yes	See d450 Walking
	USER-P	*ap_mobility_limit*					No	No T1/T4 assessment
d570 Looking after one's health	SICM III	*scim_mobility_bed*					Yes	See d410 Changing basic body position
	PASIPD	*physact_light_01*					No	No T1/T4 assessment
		physact_moderate_01					No	No T1/T4 assessment
		physact_strenuous_01					No	No T1/T4 assessment

S1 Table. Overview about the selection of relevant functioning problems and corresponding SwiSCI Inception Cohort variables according to Brief ICF Core Set for SCI (early post-acute situation)[1] and selected categories according to Ballert et al[2]. Variables selected for the network analysis are marked in green.

ICF Code	SwiSCI dataset: Instrument	SwiSCI dataset: Name of candidate SwiSCI variable	Missing observations T1, N (%): Stable high functioning class (N=239)	Missing observations T1, N (%): Moderate functioning improvement class (N=753)	Missing observations T4, N (%): Stable high functioning class (N=239)	Missing observations T4, N (%): Moderate functioning improvement class (N=753)	Inclusion decision (Yes/No)	Comment
		physact_power_01					No	No T1/T4 assessment
	ISCoS Pulmonary function basic data set	*smoking_status*					No	No T1 assessment
	SHS	*alcohol_status*					No	No T1/T4 assessment
		diet					No	No T1/T4 assessment
		drugs					No	No T1/T4 assessment
d630 Preparing meals	USER-P***							No variables identified
d770 Intimate relationships	USER-P	ap_partner_limit					No	No T1/T4 assessment
		ap_partner_sat					No	No T1/T4 assessment
	Basic socio-demo-graphics***						No	Not available in dataset**
d870 Economic self-sufficiency								No variables identified
d930 Religion and spiritualiy	USER-P	*ap_outdoor_tms*					No	No T1/T4 assessment

Variable names in italic indicate that the corresponding ICF code is only reflected in the additional concept of the variable or no information on the detailed ICF linking was available. *One out of a set of possible variables assessed with the same instrument has been tested for missing observations. It is assumed that the remaining variables show a similar missingness pattern. ** Not available in our dataset for testing missing observations. It is assumed that the corresponding variables show similar missingness patterns to similar variables or variables assessed with the same instrument. *** No information on the ICF linking available on the level of single items. Abbreviations: BPI, Brief Pain Inventory; CIRS, Cumulative Illness Rating Scale; Brief COPE, Brief Coping Orientation to Problems Experienced Inventory; GSES, General Self-Efficacy Scale; HADS, Hospital Anxiety and Depression Schedule; ICF, International Classification of Functioning, Disability and Health; IES-6, Impact of Event Scale-6; IMPACT-S, ICF Measure of Participation and ACTivities; ISCoS, International Spinal Cord Society; ISNCSCI, International Standards for Neurological Classification of Spinal Cord Injury; LOT, Life Orientation Test; MAS, Modified Ashworth Scale; NA, not available; PASIPD, Physical Activity Scale for Individuals with Physical Disabilities; RSES, Rosenberg Self-Esteem Scale; SCIM III, Spinal Cord Independence Measure version III; SCIM-SR, Spinal Cord Independence Measure for Self-Report; SCIPI, Spinal Cord Injury Pain Instrument; SCI-SCS, Spinal Cord Injury – Secondary Conditions Scale; SF-36, Short Form Health Survey, 36 Item Version; SHP, Swiss Household Panel; SHS, Swiss Health Survey; SwiSCI, Swiss Spinal Cord Injury Cohort Study; T1, SwiSCI assessment time point 1 (admission); T4, SwiSCI assessment time point 4 (discharge); USER-P, Utrecht Scale for Evaluation of Rehabilitation Participation.
