# Supplementary material for: Revisiting functioning recovery in persons with spinal cord injury undergoing first rehabilitation: Trajectory and network analysis of a Swiss cohort study
Source: PLoS One. 2024 Feb 9;19(2):e0297682. doi: 10.1371/journal.pone.0297682 (PMC10857630; doi:10.1371/journal.pone.0297682)
Supplement: S2 Table — (PDF) [file pone.0297682.s002.pdf]

S2 Table. SCIM III items and scoring after Itzkovich et al[1], study labels and corresponding scoring system and ICF domains used in the study.

SCIM III labelling			SCIM III scoring system			ICF linking				
Item name	Study label	Network analysis label	No. of response categories	Original scoring	Scoring used for Rasch analysis	Brief ICF Core Set for SCI[2]	ICF Set of Ballert et al[3]	Main ICF code*	Additional ICF code*	ICF domains used for network analysis and corresponding colour code**
Self-Care										
Feeding	Feeding	Feeding	4	0,1,2,3	original	X<br>X		d550,<br>d560		Eating / Drinking
Bathing – upper body	Bathing upper body	Bathing upper	4	0,1,2,3	original	X		d510		Washing oneself/ Caring for body parts
Bathing – lower body	Bathing lower body	Bathing lower	4	0,1,2,3	original	X		d510		Washing oneself/ Caring for body parts
Dressing – upper body	Dressing upper body	Dressing upper	5	0,1,2,3,4	original	X		d540		Dressing
Dressing – lower body	Dressing lower body	Dressing lower	5	0,1,2,3,4	original	X		d540		Dressing
Grooming	Grooming	Grooming	4	0,1,2,3	original	X		d510,<br>d520		Washing oneself/ Caring for body parts
Respiration and Sphincter Management										
Respiration	Respiration	Respiration	6	0,2,4,6,8,10	0,1,2,3,4,5	X		b440		Respiration functions
Sphincter Management – Bladder	Bladder management	Bladder mgmt.	7	0,3,6,9,11,13,15	0,1,2,3,4,5,6	X<br>X		d530	 b620	Toileting
Sphincter Management – Bowel	Bowel management	Bowel mgmt.	4	0,5,8,10	0,1,2,3	X<br>X		d530	 b525	Toileting
Use of toilet	Use of toilet	Use of toilet	5	0,1,2,4,5	0,1,2,3,4	X		d530		Toileting
Mobility – room and toilet										
Mobility in Bed and Actin to Prevent Pressure Sores	Mobility in bed	Mobility in bed	4	0,2,4,6	0,1,2,3	X	 X	d410	 d570	Changing basic body position/ Transferring oneself
Transfers: Bed-wheelchair	Transfer bed-wheelchair	Transfer bed-w/c	3	0,1,2	original	X<br>X		d410,<br>d420		Changing basic body position/ Transferring oneself
Transfers: Wheelchair-toilet-tub	Transfer wheelchair-toilet	Transfer w/c-toilet	3	0,1,2	original	X<br>X		d420	 d530	Changing basic body position/ Transferring oneself
Mobility – indoors and outdoors, on even surfaces										
Mobility Indoors	Mobility indoors	Indoor mobility	9	0,1,2,3,4,5,6,7,8	original	X	 X	d450,<br>d465		Walking/ Moving around/ Moving around using equipment
Mobility for Moderate Distances (10-100 meters)	Mobility moderate distances	Moderate mobility	9	0,1,2,3,4,5,6,7,8	original	X	 X	d450,<br>d465		Walking/ Moving around/ Moving around using equipment
Mobility Outdoors (more than 100 meters)	Mobility outdoors	Outdoor mobility	9	0,1,2,3,4,5,6,7,8	original	X	 X	d450,<br>d465		Walking/ Moving around/ Moving around using equipment
Stair Management	Stair management	Stairs mgmt.	4	0,1,2,3	original			d455		Walking/ Moving around/ Moving around using equipment
Transfers: Wheelchair-car	Transfer wheelchair-car	Transfer w/c-car	3	0,1,2	original	X		d420		Changing basic body position/ Transferring oneself
Transfers: Ground-wheelchair	Transfer ground-wheelchair	Transfer ground-w/c	2	0,1	original	X		d420		Changing basic body position/ Transferring oneself

*All ICF codes were considered at second level; **Domains were summarized based on main ICF codes of SCIM III items. Abbreviations: ICF, International Classification of Functioning, Disability and Health; SCIM III, Spinal Cord Independence Measure version III.

[1] Itzkovich M, Gelernter I, Biering-Sørensen F, Weeks C, Laramee MT, Craven BC, et al. The Spinal Cord Independence Measure (SCIM) version III: Reliability and validity in a multi-center international study. Disabil Rehabil 2007; 29: 1926-1933.

[2] Kirchberger I, Cieza A, Biering-Sørensen F, Baumberger M, Charlifue S, Post MW et al. ICF Core Sets for individuals with spinal cord injury in the early post-acute context. Spinal Cord 2010;48(4):297-304.

[3] Ballert C, Oberhauser C, Biering-Sørensen F, Stucki G, Cieza A. Explanatory power does not equal clinical importance: study of the use of the Brief ICF Core Sets for Spinal Cord Injury with a purely statistical approach. Spinal Cord 2012;50(10):734-9.
