# Supplementary material for: Revisiting functioning recovery in persons with spinal cord injury undergoing first rehabilitation: Trajectory and network analysis of a Swiss cohort study
Source: PLoS One. 2024 Feb 9;19(2):e0297682. doi: 10.1371/journal.pone.0297682 (PMC10857630; doi:10.1371/journal.pone.0297682)
Supplement: S3 Table — (PDF) [file pone.0297682.s003.pdf]

S4 Table. Expanded overview of available individuals who have consented to either the SwiSCI Inception Cohort or the use of routing clinical data for research purposes (MDS), as well as the excluded and included individuals for this study.

Characteristics	SwiSCI Inception Cohort or MDS (N = 1488)	Excluded from present study (N = 389)	Included within present study (N = 1099)	P-value
Sex = Female, n (%)	481 (32.33)	126 (32.39)	355 (32.30)	1.000
Age at SCI diagnosis in years, median [IQR]	58.00 [43.00, 71.00]	60.00 [45.00, 72.00]	58.00 [42.00, 70.00]	0.081
Etiology = Traumatic, n (%)	847 (56.92)	224 (57.58)	623 (56.69)	0.805
Level of injury at T1, n (%)				<0.001
Tetraplegia	489 (32.86)	129 (33.16)	360 (32.76)	
Paraplegia	736 (49.46)	135 (34.70)	601 (54.69)	
Intact	9 (0.60)	2 (0.51)	7 (0.64)	
Missing	254 (17.07)	123 (31.62)	131 (11.92)	
Level of injury at T4, n (%)				<0.001
Tetraplegia	458 (30.78)	127 (32.65)	331 (30.12)	
Paraplegia	759 (51.01)	164 (42.16)	595 (54.14)	
Intact	30 (2.02)	12 (3.08)	18 (1.64)	
Missing	241 (16.20)	86 (22.11)	155 (14.10)	
Severity of injury at T1, n (%)				<0.001
AIS A	285 (19.15)	72 (18.51)	213 (19.38)	
AIS B	142 (9.54)	28 (7.20)	114 (10.37)	
AIS C	174 (11.69)	47 (12.08)	127 (11.56)	
AIS D	619 (41.60)	119 (30.59)	500 (45.50)	
AIS E	8 (0.54)	2 (0.51)	6 (0.55)	
Missing	260 (17.47)	121 (31.11)	139 (12.65)	
Severity of injury at T4, n (%)				<0.001
AIS A	245 (16.47)	71 (18.25)	174 (15.83)	
AIS B	93 (6.25)	13 (3.34)	80 (7.28)	
AIS C	108 (7.26)	23 (5.91)	85 (7.73)	
AIS D	755 (50.74)	180 (46.27)	575 (52.32)	
AIS E	29 (1.95)	12 (3.08)	17 (1.55)	
Missing	258 (17.34)	90 (23.14)	168 (15.29)	
Length of stay in days, median [IQR]	135.00 [74.00, 191.00]	124.50 [49.00, 195.00]	139.00 [80.00, 190.00]	0.001
Missing, n (%)	3 (0.20)	3 (0.77)	0 (0.00)	
Interval-based SCIM III sum score at T1, median [IQR]	74.60 [58.33, 87.42]	40.25 [12.30, 68.41]	75.93 [62.75, 87.77]	<0.001
Missing, n (%)	317 (21.30)	264 (67.87)	53 (4.82)	
Interval-based SCIM III sum score at T2, median [IQR]	85.98 [74.60, 94.07]	68.41 [50.38, 83.84]	86.97 [77.20, 94.42]	<0.001
Missing, n (%)	911 (61.22)	341 (87.66)	570 (51.87)	

S4 Table. Expanded overview of available individuals who have consented to either the SwiSCI Inception Cohort or the use of routing clinical data for research purposes (MDS), as well as the excluded and included individuals for this study.

Characteristics	SwiSCI Inception Cohort or MDS (N = 1488)	Excluded from present study (N = 389)	Included within present study (N = 1099)	P-value
Interval-based SCIM III sum score at T3, median [IQR]	84.92 [71.67, 91.72]	80.45 [56.50, 84.77]	86.46 [74.60, 92.70]	0.002
Missing, n (%)	1196 (80.38)	351 (90.23)	845 (76.89)	
Interval-based SCIM III sum score at T4, median [IQR]	94.42 [84.92, 97.30]	90.98 [75.93, 97.27]	94.42 [85.98, 97.30]	0.002
Missing, n (%)	204 (13.71)	192 (49.36)	12 (1.09)	
Assessment time point SCIM III T1 in days, median [IQR]	9.00 [1.00, 19.00]	-8.50 [-21.25, 1.00]	11.00 [2.00, 19.00]	<0.001
Missing, n (%)	314 (21.10)	261 (67.10)	53 (4.82)	
Assessment time point SCIM III T2 in days, median [IQR]	67.00 [55.00, 76.00]	44.00 [17.75, 54.75]	69.00 [57.00, 77.00]	<0.001
Missing, n (%)	911 (61.22)	341 (87.66)	570 (51.87)	
Assessment time point SCIM III T3 in days, median [IQR]	145.00 [131.00, 158.00]	129.00 [111.25, 140.75]	147.00 [134.00, 159.00]	<0.001
Missing, n (%)	1196 (80.38)	351 (90.23)	845 (76.89)	
Assessment time point SCIM III T4 in days, median [IQR]	133.00 [71.25, 188.00]	125.00 [34.50, 201.50]	135.00 [76.00, 186.50]	0.060
Missing, n (%)	202 (13.58)	190 (48.84)	12 (1.09)	
Language of correspondence, n (%)				0.033
German	1128 (75.81)	314 (80.72)	814 (74.07)	
French	291 (19.56)	60 (15.42)	231 (21.02)	
Italian	42 (2.82)	11 (2.83)	31 (2.82)	
Other	18 (1.21)	1 (0.26)	17 (1.55)	
Missing	9 (0.60)	3 (0.77)	6 (0.55)	
Insurance type, n (%)				<0.001
Health	826 (55.51)	137 (35.22)	689 (62.69)	
Disability	8 (0.54)	0 (0.00)	8 (0.73)	
Military	1 (0.07)	0 (0.00)	1 (0.09)	
Accident	471 (31.65)	81 (20.82)	390 (35.49)	
Other	1 (0.07)	0 (0.00)	1 (0.09)	
Self-pay	1 (0.07)	1 (0.26)	0 (0.00)	
Missing	180 (12.10)	170 (43.70)	10 (0.91)	
Ward type, n (%)				<0.001
Basic	541 (36.36)	84 (21.59)	457 (41.58)	
Semi-private	291 (19.56)	55 (14.14)	236 (21.47)	
Private	154 (10.35)	30 (7.71)	124 (11.28)	
Missing	502 (33.74)	220 (56.56)	282 (25.66)	
Partner at time of SCI diagnosis, n (%)				<0.001
No	214 (14.38)	27 (6.94)	187 (17.02)	

S4 Table. Expanded overview of available individuals who have consented to either the SwiSCI Inception Cohort or the use of routing clinical data for research purposes (MDS), as well as the excluded and included individuals for this study.

Characteristics	SwiSCI Inception Cohort or MDS (N = 1488)	Excluded from present study (N = 389)	Included within present study (N = 1099)	P-value
Yes	459 (30.85)	40 (10.28)	419 (38.13)	
Missing	815 (54.77)	322 (82.78)	493 (44.86)	
Cardiovascular complications at T1, n (%)				<0.001
No	831 (55.85)	121 (31.11)	710 (64.60)	
Yes	464 (31.18)	79 (20.31)	385 (35.03)	
Missing	193 (12.97)	189 (48.59)	4 (0.36)	
Pulmonary complications at T1, n (%)				<0.001
No	832 (55.91)	93 (23.91)	739 (67.24)	
Yes	452 (30.38)	100 (25.71)	352 (32.03)	
Missing	204 (13.71)	196 (50.39)	8 (0.73)	
Ventilation assistance at T1, n (%)				<0.001
No	1116 (75.00)	138 (35.48)	978 (88.99)	
Yes	165 (11.09)	55 (14.14)	110 (10.01)	
Missing	207 (13.91)	196 (50.39)	11 (1.00)	
Normal defecation at T1, n (%)				<0.001
No	463 (31.12)	41 (10.54)	422 (38.40)	
Yes	219 (14.72)	18 (4.63)	201 (18.29)	
Missing	806 (54.17)	330 (84.83)	476 (43.31)	
Urinary tract infection at T1, n (%)				<0.001
No	596 (40.05)	77 (19.79)	519 (47.22)	
Yes	256 (17.20)	16 (4.11)	240 (21.84)	
Missing	636 (42.74)	296 (76.09)	340 (30.94)	
Pressure injury at T1, n (%)				<0.001
No	661 (44.42)	72 (18.51)	589 (53.59)	
Yes	201 (13.51)	22 (5.66)	179 (16.29)	
Missing	626 (42.07)	295 (75.84)	331 (30.12)	
Pain at T1, n (%)				<0.001
No	127 (8.53)	4 (1.03)	123 (11.19)	
Yes	348 (23.39)	21 (5.40)	327 (29.75)	
Missing	1013 (68.08)	364 (93.57)	649 (59.05)	

Variable distributions between excluded and included samples were compared using p-values of the Mann-Whitney-U-Test for continuous variables and the Pearson's Chi-square test for categorical variables (both with continuity correction). Abbreviations: AIS, American Spinal Injury Association Impairment Scale; IQR, Interquartile range; SCIM III, Spinal Cord Independence Measure version III; SwiSCI, Swiss Spinal Cord Injury Cohort Study; T1-T4, SwiSCI assessment time points 1-4.
