# Supplementary material for: Revisiting functioning recovery in persons with spinal cord injury undergoing first rehabilitation: Trajectory and network analysis of a Swiss cohort study
Source: PLoS One. 2024 Feb 9;19(2):e0297682. doi: 10.1371/journal.pone.0297682 (PMC10857630; doi:10.1371/journal.pone.0297682)
Supplement: S4 Table — (PDF) [file pone.0297682.s004.pdf]

S5 Table. Main results of the Rasch analysis of the SCIM III total score.

Overall item and person fit		Individual item fit	Item-trait interaction	Reliability		Unidimensionality	Local independence	Group invariance	Stochastic ordering
Mean item fit residual (SD)	Mean person fit residual (SD)	Non-significant Bonferroni corrected p-values for all items (yes/no)	Chi-squared test statistic (df, p-value)	PSI	Alpha	Lower bound of CI for paired t-test	Item independence according to residual correlation matrix (yes/no)	Presence of DIF (item number)	Ordered item thresholds (yes/no)
Baseline analysis based on calibration sample (N=400, number of class intervals=6)									
0.235 (3.322)	0.142 (1.203)	no	3838.9 (95, 0.000)	0.966	0.917	21.9%	no	Etiology (1),<br>Age (3,5,6,15,19),<br>Timepoint (6,13)<br>Center (3,11,12,13,14,17)	no
Two testlet approach based on calibration sample (N=400, number of class intervals=6)									
0.914 (0.761)	-0.376 (0.677)	yes	16.3 (10,0.092)	0.921	0.822	4.6%	yes	Age (1,2),*<br>Timepoint (2),*<br>Center (1)*	NA
Two testlet approach based on validation sample (N=400, number of class intervals=6)									
0.765 (0.764)	-0.429 (0.811)	yes	14.8 (0.141)	0.877	0.809	4.3%	yes	Timepoint (1,2)*<br>Center (1,2)*	NA

Indication of good fit is as follows: Mean item and person fit residual's SD<1.4 for overall item and person fit; Non-significant Bonferroni-corrected p-values of individual item fit residuals; non-significant p-value of the chi-squared test statistic for the item-trait interaction; PSI>0.7 and alpha>0.7 for reliability; Lower bound of CI for paired t-test<5% for unidimiensionality; Presence of local independence of items; No DIF present for group invariance; Ordered item thresholds for stochastic ordering. DIF was tested for age groups (16-30 years, 31-45 years, 46-60 years, 61-75 years, 76+ years), SwiSCI study centers (Balgrist, Basel, Nottwil, Sion), etiology (traumatic, non-traumatic), sex (male, female), and SwiSCI time points (T1, T2, T3, T4). *Marginal DIF (<0.5 logits difference between mean locations per class interval). Abbreviations: CI, confidence interval; df, degrees of freedom; DIF, Differential Item Functioning; NA, not applicable; PSI, Person Separation Index; SCIM III, Spinal Cord Independence Measure version III; SD, standard deviation; SwiSCI, Swiss Spinal Cord Injury Cohort Study.
