# Supplementary material for: Revisiting functioning recovery in persons with spinal cord injury undergoing first rehabilitation: Trajectory and network analysis of a Swiss cohort study
Source: PLoS One. 2024 Feb 9;19(2):e0297682. doi: 10.1371/journal.pone.0297682 (PMC10857630; doi:10.1371/journal.pone.0297682)
Supplement: S5 Table — (PDF) [file pone.0297682.s005.pdf]

S10 Table. Posterior classification table of the best-fitting latent process mixed model.

Classes	N (%)	Mean posterior class membership probabilities in percentage			
		Stable high functioning	Early functioning improvement	Moderate functioning improvement	Slow functioning improvement
Stable high functioning	239 (21.75)	84.69	0.44	14.87	0.00
Early functioning improvement	33 (3.00)	0.04	78.91	21.05	0.00
Moderate functioning improvement	753 (68.52)	3.06	3.90	91.30	1.75
Slow functioning improvement	74 (6.73)	0.00	0.01	15.81	84.18
