# Supplementary material for: Revisiting functioning recovery in persons with spinal cord injury undergoing first rehabilitation: Trajectory and network analysis of a Swiss cohort study
Source: PLoS One. 2024 Feb 9;19(2):e0297682. doi: 10.1371/journal.pone.0297682 (PMC10857630; doi:10.1371/journal.pone.0297682)
Supplement: S6 Table — (PDF) [file pone.0297682.s006.pdf]

S11 Table. Estimated parameters of the best-fitting latent process mixed model.

A) Fixed effects in the class-membership model (reference=class 4)

Parameter	Coefficient	SE	P-value
Intercept class 1	-2.57578	0.33038	0.00000
Intercept class 2	-1.19080	0.11239	0.00000
Intercept class 3	-2.28487	0.21872	0.00000

Note: Class 1, early functioning improvement class; class 2, stable high functioning class; class 3 slow functioning improvement class; class 4, moderate functioning improvement class.
Abbreviations: SE, standard error.

B) Fixed effects of the longitudinal model

Parameter	Coefficient	SE	P-value
Intercept class 1 (not estimated)	0		
Intercept class 2	5.74738	0.53073	0.00000
Intercept class 3	-2.99606	0.54428	0.00000
Intercept class 4	0.93008	0.54400	0.08732
Time of assessment Class 1	0.06250	0.00451	0.00000
Time of assessment Class 2	0.00746	0.00143	0.00000
Time of assessment Class 3	0.00814	0.00119	0.00000
Time of assessment Class 4	0.02293	0.00085	0.00000

Note: Class 1, early functioning improvement class; class 2, stable high functioning class; class 3 slow functioning improvement class; class 4, moderate functioning improvement class.
Abbreviations: SE, standard error.

C) Variance-covariance matrix of the random effects for class 4

	Intercept	Time of assessment
Intercept	3.91802	
Time of assessment	-0.00052	0.00007

Note: Class 4, moderate functioning improvement class.

D) Proportional coefficients for variance-covariance matrix of the random effects

	Coefficient	SE
Class 1	0.82651	0.15679
Class 2	0.00001	0.03366
Class 3	0.36224	0.09393
Residual SE (not estimated)	1	-

Note: Class 1, early functioning improvement class; class 2, stable high functioning class; class 3 slow functioning improvement class.
Abbreviations: SE, standard error.

E) Parameters of the link function (quadratic I-splines with two knots)

Parameter	Coefficient	SE	P-value
I-splines1	-4.60805	0.54471	0.00000
I-splines2	1.27000	0.07808	0.00000
I-splines3	0.00014	0.02845	0.99619
I-splines4	3.16304	0.03736	0.00000

Abbreviations: SE, standard error.
