# Supplementary material for: Revisiting functioning recovery in persons with spinal cord injury undergoing first rehabilitation: Trajectory and network analysis of a Swiss cohort study
Source: PLoS One. 2024 Feb 9;19(2):e0297682. doi: 10.1371/journal.pone.0297682 (PMC10857630; doi:10.1371/journal.pone.0297682)
Supplement: S7 Table — (PDF) [file pone.0297682.s007.pdf]

S12 Table. Extended overview of study participant characteristics for the identified classes of functioning trajectories.

Characteristics	Stable high functioning class (N = 239)	Early functioning improvement class (N = 33)	Moderate functioning improvement class (N = 753)	Slow functioning improvement class (N = 74)
Sex = Female, n (%)	78 (32.64)	12 (36.36)	251 (33.33)	14 (18.92)
Age at SCI diagnosis in years, median [IQR]	51.00 [37.00, 60.50]	61.00 [46.00, 69.00]	60.00 [44.00, 72.00]	65.50 [43.25, 74.00]
Etiology = Traumatic, n (%)	132 (55.23)	22 (66.67)	426 (56.57)	43 (58.11)
Level of injury at T1, n (%)				
Tetraplegia	71 (29.71)	16 (48.48)	211 (28.02)	62 (83.78)
Paraplegia	121 (50.63)	9 (27.27)	466 (61.89)	5 (6.76)
Intact	5 (2.09)	0 (0.00)	2 (0.27)	0 (0.00)
Missing	42 (17.57)	8 (24.24)	74 (9.83)	7 (9.46)
Level of injury at T4, n (%)				
Tetraplegia	63 (26.36)	15 (45.45)	195 (25.90)	58 (78.38)
Paraplegia	120 (50.21)	13 (39.39)	458 (60.82)	4 (5.41)
Intact	10 (4.18)	1 (3.03)	7 (0.93)	0 (0.00)
Missing	46 (19.25)	4 (12.12)	93 (12.35)	12 (16.22)
Severity of injury at T1, n (%)				
AIS A	5 (2.09)	1 (3.03)	178 (23.64)	29 (39.19)
AIS B	9 (3.77)	0 (0.00)	93 (12.35)	12 (16.22)
AIS C	4 (1.67)	5 (15.15)	98 (13.01)	20 (27.03)
AIS D	172 (71.97)	20 (60.61)	302 (40.11)	6 (8.11)
AIS E	4 (1.67)	0 (0.00)	2 (0.27)	0 (0.00)
Missing	45 (18.83)	7 (21.21)	80 (10.62)	7 (9.46)
Severity of injury at T4, n (%)				
AIS A	4 (1.67)	1 (3.03)	147 (19.52)	22 (29.73)
AIS B	9 (3.77)	0 (0.00)	62 (8.23)	9 (12.16)
AIS C	0 (0.00)	0 (0.00)	66 (8.76)	19 (25.68)
AIS D	166 (69.46)	27 (81.82)	371 (49.27)	11 (14.86)
AIS E	9 (3.77)	1 (3.03)	7 (0.93)	0 (0.00)
Missing	51 (21.34)	4 (12.12)	100 (13.28)	13 (17.57)
Length of stay in days, median [IQR]	59.00 [42.00, 85.50]	105.00 [92.00, 128.00]	165.00 [113.00, 201.00]	246.50 [190.50, 276.50]
Interval-based SCIM III sum score at T1, median [IQR]	95.96 [94.07, 97.30]	62.75 [50.38, 68.41]	73.18 [62.75, 81.39]	36.29 [27.02, 40.25]
Missing, n (%)	0 (0.00)	7 (9.46)	32 (4.25)	14 (5.86)
Interval-based SCIM III sum score at T2, median [IQR]	97.27 [95.96, 98.31]	94.95 [91.00, 97.30]	85.98 [78.36, 91.53]	43.91 [36.29, 53.23]
Missing, n (%)	14 (42.42)	31 (41.89)	343 (45.55)	182 (76.15)
Interval-based SCIM III sum score at T3, median [IQR]	96.97 [96.14, 98.54]	95.87 [94.97, 96.77]	88.33 [78.36, 94.07]	50.38 [43.91, 60.61]

S12 Table. Extended overview of study participant characteristics for the identified classes of functioning trajectories.

Characteristics	Stable high functioning class (N = 239)	Early functioning improvement class (N = 33)	Moderate functioning improvement class (N = 753)	Slow functioning improvement class (N = 74)
Missing, n (%)	31 (93.94)	33 (44.59)	547 (72.64)	234 (97.91)
Interval-based SCIM III sum score at T4, median [IQR]	98.54 [97.27, 99.62]	97.58 [96.01, 98.93]	91.72 [85.45, 95.96]	54.56 [43.91, 62.75]
Missing, n (%)	1 (3.03)	0 (0.00)	7 (0.93)	4 (1.67)
Assessment time point SCIM III T1 in days, median [IQR]	10.00 [2.00, 20.00]	7.00 [2.00, 17.00]	11.00 [2.00, 19.00]	14.00 [5.00, 23.00]
Missing, n (%)	0 (0.00)	7 (9.46)	32 (4.25)	14 (5.86)
Assessment time point SCIM III T2 in days, median [IQR]	56.00 [43.00, 71.00]	69.00 [61.50, 78.50]	69.00 [59.00, 77.00]	71.00 [62.50, 82.00]
Missing, n (%)	14 (42.42)	31 (41.89)	343 (45.55)	182 (76.15)
Assessment time point SCIM III T3 in days, median [IQR]	91.00 [27.00, 148.00]	136.50 [134.25, 138.75]	147.00 [134.25, 159.00]	150.00 [139.00, 161.00]
Missing, n (%)	31 (93.94)	33 (44.59)	547 (72.64)	234 (97.91)
Assessment time point SCIM III T4 in days, median [IQR]	55.00 [38.50, 82.50]	102.00 [91.00, 126.75]	161.00 [108.00, 195.00]	243.00 [187.25, 268.75]
Missing, n (%)	1 (3.03)	0 (0.00)	7 (0.93)	4 (1.67)
Associated injuries, n (%)				
No	77 (32.22)	8 (24.24)	175 (23.24)	16 (21.62)
Yes	56 (23.43)	18 (54.55)	311 (41.30)	51 (68.92)
Missing	106 (44.35)	7 (21.21)	267 (35.46)	7 (9.46)
Comorbidities before SCI diagnosis, n (%)				
No	45 (18.83)	9 (27.27)	118 (15.67)	13 (17.57)
Yes	186 (77.82)	24 (72.73)	615 (81.67)	58 (78.38)
Missing	8 (3.35)	0 (0.00)	20 (2.66)	3 (4.05)
Language of correspondence, n (%)				
German	177 (74.06)	23 (69.70)	563 (74.77)	51 (68.92)
French	56 (23.43)	8 (24.24)	152 (20.19)	15 (20.27)
Italian	3 (1.26)	2 (6.06)	23 (3.05)	3 (4.05)
Other	2 (0.84)	0 (0.00)	12 (1.59)	3 (4.05)
Missing	1 (0.42)	0 (0.00)	3 (0.40)	2 (2.70)
Insurance type, n (%)				
Health	173 (72.38)	21 (63.64)	462 (61.35)	33 (44.59)
Disability	1 (0.42)	0 (0.00)	6 (0.80)	1 (1.35)
Military	0 (0.00)	0 (0.00)	1 (0.13)	0 (0.00)
Accident	63 (26.36)	12 (36.36)	276 (36.65)	39 (52.70)
Other	0 (0.00)	0 (0.00)	1 (0.13)	0 (0.00)
Missing	2 (0.84)	0 (0.00)	7 (0.93)	1 (1.35)
Ward type, n (%)				

S12 Table. Extended overview of study participant characteristics for the identified classes of functioning trajectories.

Characteristics	Stable high functioning class (N = 239)	Early functioning improvement class (N = 33)	Moderate functioning improvement class (N = 753)	Slow functioning improvement class (N = 74)
Basic	126 (52.72)	15 (45.45)	293 (38.91)	23 (31.08)
Semi-private	47 (19.67)	2 (6.06)	169 (22.44)	18 (24.32)
Private	22 (9.21)	5 (15.15)	87 (11.55)	10 (13.51)
Missing	44 (18.41)	11 (33.33)	204 (27.09)	23 (31.08)
Partner at time of SCI diagnosis, n (%)				
No	44 (18.41)	3 (9.09)	131 (17.40)	9 (12.16)
Yes	102 (42.68)	17 (51.52)	277 (36.79)	23 (31.08)
Missing	93 (38.91)	13 (39.39)	345 (45.82)	42 (56.76)
Cardiovascular complications at T1, n (%)				
No	183 (76.57)	21 (63.64)	466 (61.89)	40 (54.05)
Yes	54 (22.59)	12 (36.36)	285 (37.85)	34 (45.95)
Missing	2 (0.84)	0 (0.00)	2 (0.27)	0 (0.00)
Pulmonary complications at T1, n (%)				
No	209 (87.45)	22 (66.67)	484 (64.28)	24 (32.43)
Yes	27 (11.30)	11 (33.33)	264 (35.06)	50 (67.57)
Missing	3 (1.26)	0 (0.00)	5 (0.66)	0 (0.00)
Ventilation assistance at T1, n (%)				
No	233 (97.49)	30 (90.91)	671 (89.11)	44 (59.46)
Yes	2 (0.84)	3 (9.09)	75 (9.96)	30 (40.54)
Missing	4 (1.67)	0 (0.00)	7 (0.93)	0 (0.00)
Normal defecation at T1, n (%)				
No	40 (16.74)	13 (39.39)	334 (44.36)	35 (47.30)
Yes	107 (44.77)	7 (21.21)	86 (11.42)	1 (1.35)
Missing	92 (38.49)	13 (39.39)	333 (44.22)	38 (51.35)
Urinary tract infection at T1, n (%)				
No	145 (60.67)	18 (54.55)	322 (42.76)	34 (45.95)
Yes	21 (8.79)	7 (21.21)	195 (25.90)	17 (22.97)
Missing	73 (30.54)	8 (24.24)	236 (31.34)	23 (31.08)
Pressure injury at T1, n (%)				
No	164 (68.62)	20 (60.61)	374 (49.67)	31 (41.89)
Yes	2 (0.84)	4 (12.12)	151 (20.05)	22 (29.73)
Missing	73 (30.54)	9 (27.27)	228 (30.28)	21 (28.38)
Pain at T1, n (%)				

S12 Table. Extended overview of study participant characteristics for the identified classes of functioning trajectories.

Characteristics	Stable high functioning class (N = 239)	Early functioning improvement class (N = 33)	Moderate functioning improvement class (N = 753)	Slow functioning improvement class (N = 74)
No	40 (16.74)	4 (12.12)	77 (10.23)	2 (2.70)
Yes	80 (33.47)	10 (30.30)	225 (29.88)	12 (16.22)
Missing	119 (49.79)	19 (57.58)	451 (59.89)	60 (81.08)

Abbreviations: AIS, American Spinal Injury Association Impairment Scale; SCIM III, Spinal Cord Independence Measure version III; SwiSCI, Swiss Spinal Cord Injury Cohort Study; T1-T4, SwiSCI assessment time points 1-4.
