# Supplementary material for: Revisiting functioning recovery in persons with spinal cord injury undergoing first rehabilitation: Trajectory and network analysis of a Swiss cohort study
Source: PLoS One. 2024 Feb 9;19(2):e0297682. doi: 10.1371/journal.pone.0297682 (PMC10857630; doi:10.1371/journal.pone.0297682)
Supplement: S8 Table — A) T1. B) T4. (PDF) [file pone.0297682.s008.pdf]

**S13 Table. Edge weights of estimated mixed graphical model network for the moderate functioning improvement class.**

A) T1.

| Connecting node 1 | Connecting node 2 | Edge weight |
|---|---|---|
| Mobility indoors | Mobility moderate distances | 0.635 |
| Transfer bed-wheelchair | Transfer wheelchair-toilet | 0.499 |
| Bathing upper body | Dressing upper body | 0.460 |
| Mobility moderate distances | Mobility outdoors | 0.390 |
| Bathing lower body | Dressing lower body | 0.334 |
| Injury level | Feeding | 0.330 |
| Feeding | Grooming | 0.319 |
| Dressing lower body | Use of toilet | 0.266 |
| Transfer wheelchair-toilet | Transfer wheelchair-car | 0.257 |
| Age | Injury Severity | 0.215 |
| Stair management | Transfer ground-wheelchair | 0.214 |
| Stair management | Transfer wheelchair-car | 0.165 |
| Bathing lower body | Use of toilet | 0.164 |
| Dressing upper body | Mobility in bed | 0.163 |
| Injury Severity | Mobility in bed | 0.161 |
| Feeding | Dressing upper body | 0.155 |
| Dressing upper body | Grooming | 0.148 |
| Use of toilet | Mobility in bed | 0.145 |
| Bladder management | Bowel management | 0.141 |
| Dressing lower body | Mobility in bed | 0.120 |
| Bathing upper body | Grooming | 0.118 |
| Mobility in bed | Transfer bed-wheelchair | 0.108 |
| Mobility indoors | Mobility outdoors | 0.108 |
| Age | Sex | 0.098 |
| Feeding | Bathing upper body | 0.081 |
| Bathing lower body | Mobility in bed | 0.080 |
| Injury Severity | Use of toilet | 0.067 |
| Bathing upper body | Bathing lower body | 0.064 |
| Bowel management | Use of toilet | 0.060 |
| Dressing upper body | Mobility indoors | 0.059 |
| Use of toilet | Stair management | 0.059 |
| Use of toilet | Transfer wheelchair-toilet | 0.055 |
| Grooming | Respiration | 0.054 |
| Injury level | Grooming | 0.051 |
| Dressing upper body | Use of toilet | 0.036 |
| Age | Bathing lower body | -0.067 |
| Injury level | Injury Severity | -0.091 |

Abbreviation: T1, Swiss Spinal Cord Injury Cohort Study assessment time point 1.

B) T4.

| Connecting node 1 | Connecting node 2 | Edge weight |
|---|---|---|
| Mobility indoors | Mobility moderate distances | 0.559 |
| Feeding | Grooming | 0.430 |
| Bathing lower body | Dressing lower body | 0.361 |
| Bowel management | Use of toilet | 0.34 |
| Mobility moderate distances | Mobility outdoors | 0.309 |
| Mobility moderate distances | Stair management | 0.286 |
| Age | Injury Severity | 0.275 |
| Transfer wheelchair-toilet | Transfer wheelchair-car | 0.271 |
| Bathing upper body | Bathing lower body | 0.267 |
| Stair management | Transfer ground-wheelchair | 0.267 |
| Bathing lower body | Use of toilet | 0.266 |
| Injury Severity | Stair management | 0.244 |
| Bathing upper body | Dressing upper body | 0.233 |
| Dressing upper body | Dressing lower body | 0.230 |
| Mobility in bed | Transfer bed-wheelchair | 0.225 |
| Mobility indoors | Stair management | 0.213 |
| Transfer bed-wheelchair | Transfer wheelchair-car | 0.192 |
| Use of toilet | Mobility in bed | 0.176 |
| Feeding | Dressing upper body | 0.173 |
| Dressing lower body | Mobility in bed | 0.144 |
| Dressing lower body | Use of toilet | 0.134 |
| Dressing lower body | Transfer ground-wheelchair | 0.131 |
| Injury level | Feeding | 0.120 |
| Mobility outdoors | Transfer wheelchair-car | 0.113 |
| Mobility outdoors | Transfer ground-wheelchair | 0.109 |
| Transfer bed-wheelchair | Transfer wheelchair-toilet | 0.108 |
| Bladder management | Use of toilet | 0.103 |
| Injury level | Dressing upper body | 0.094 |
| Age | Sex | 0.093 |
| Bladder management | Bowel management | 0.092 |
| Bladder management | Mobility in bed | 0.090 |
| Bathing upper body | Use of toilet | 0.081 |
| Dressing upper body | Mobility in bed | 0.080 |
| Bathing upper body | Grooming | 0.076 |
| Feeding | Bathing upper body | 0.076 |
| Transfer bed-wheelchair | Mobility outdoors | 0.074 |
| Bathing lower body | Dressing upper body | 0.063 |
| Bowel management | Mobility in bed | 0.049 |
| Use of toilet | Stair management | 0.049 |
| Bathing lower body | Mobility in bed | 0.048 |
| Age | Transfer wheelchair-car | -0.087 |
| Age | Transfer ground-wheelchair | -0.122 |

Abbreviation: T4, Swiss Spinal Cord Injury Cohort Study assessment time point 4.
