# Supplementary material for: Revisiting functioning recovery in persons with spinal cord injury undergoing first rehabilitation: Trajectory and network analysis of a Swiss cohort study
Source: PLoS One. 2024 Feb 9;19(2):e0297682. doi: 10.1371/journal.pone.0297682 (PMC10857630; doi:10.1371/journal.pone.0297682)
Supplement: S9 Table — A) T1. B) T4. (PDF) [file pone.0297682.s009.pdf]

S15 Table. Expected influence (z-scores) based on the mixed graphical model networks for the moderate improvement class.

A) T1.

Node	Sample estimate	Bootstrap mean estimate	Bootstrap lower limit of 95% CI	Bootstrap upper limit of 95% CI
Mobility moderate distances	1.61	1.56	1.141	2.001
Dressing upper body	1.59	1.44	0.965	1.959
Feeding	1.14	1.04	0.558	1.582
Use of toilet	1.03	0.80	0.062	1.536
Transfer wheelchair-toilet	0.90	0.90	0.424	1.523
Mobility indoors	0.87	0.74	0.197	1.308
Mobility in bed	0.79	0.90	0.292	1.587
Bathing upper body	0.60	0.48	0.045	0.923
Dressing lower body	0.60	0.70	0.176	1.350
Grooming	0.49	0.23	-0.245	0.758
Transfer bed-wheelchair	0.22	0.54	-0.051	1.210
Bathing lower body	0.11	0.30	-0.284	0.850
Mobility outdoors	-0.14	-0.29	-0.748	0.156
Stair management	-0.34	-0.48	-1.301	0.285
Transfer wheelchair-car	-0.4	-0.24	-0.850	0.452
Injury severity	-0.63	-0.48	-1.176	0.287
Injury level	-0.83	-0.83	-1.358	-0.304
Age	-0.98	-1.08	-1.661	-0.419
Transfer ground-wheelchair	-1.08	-1.23	-1.806	-0.613
Bowel management	-1.13	-1.01	-1.550	-0.515
Bladder management	-1.33	-1.11	-1.625	-0.491
Sex	-1.47	-1.34	-1.940	-0.529
Respiration	-1.62	-1.53	-2.051	-0.988

Abbreviation: CI, confidence interval; T1, Swiss Spinal Cord Injury Cohort Study assessment time point 1.

B) T4.

Node	Sample estimate	Bootstrap mean estimate	Bootstrap lower limit of 95% CI	Bootstrap upper limit of 95% CI
Mobility moderate distances	1.60	1.55	1.169	1.957
Use of toilet	1.58	1.34	0.768	1.906
Stair management	1.31	1.22	0.807	1.677
Bathing lower body	1.16	1.07	0.658	1.491
Dressing lower body	1.14	1.10	0.643	1.536
Dressing upper body	0.77	0.79	0.285	1.313
Mobility in bed	0.59	0.61	0.119	1.098
Feeding	0.55	0.45	-0.012	0.843
Mobility indoors	0.47	0.50	0.142	1.079
Bathing upper body	0.36	0.21	-0.164	0.564
Mobility outdoors	-0.02	-0.02	-0.418	0.386
Transfer bed-wheelchair	-0.04	-0.03	-0.479	0.404
Injury severity	-0.28	-0.13	-0.608	0.464
Grooming	-0.31	-0.27	-0.664	0.072
Transfer wheelchair-car	-0.36	-0.11	-0.635	0.430
Bowel management	-0.38	-0.22	-0.626	0.241
Transfer ground-wheelchair	-0.67	-0.63	-1.060	-0.200
Transfer wheelchair-toilet	-0.68	-0.48	-0.860	0.001
Bladder management	-0.96	-0.81	-1.345	-0.310
Injury level	-1.17	-1.15	-1.607	-0.640
Age	-1.33	-1.72	-2.182	-1.224
Sex	-1.52	-1.54	-1.906	-1.061
Respiration	-1.80	-1.76	-2.065	-1.418

Abbreviation: CI, confidence interval; T4, Swiss Spinal Cord Injury Cohort Study assessment time point 4.
