# Supplementary material for: Revisiting functioning recovery in persons with spinal cord injury undergoing first rehabilitation: Trajectory and network analysis of a Swiss cohort study
Source: PLoS One. 2024 Feb 9;19(2):e0297682. doi: 10.1371/journal.pone.0297682 (PMC10857630; doi:10.1371/journal.pone.0297682)
Supplement: S10 Table — A) T1. B) T4. (PDF) [file pone.0297682.s010.pdf]

S16 Table. Bridge expected influence (z-scores) based on the mixed graphical model networks for the moderate improvement class.

A) T1.

Node	Sample estimate	Bootstrap mean estimate	Bootstrap lower limit of 95% CI	Bootstrap upper limit of 95% CI
Dressing upper body	2.09	1.95	1.534	2.385
Feeding	1.67	1.60	1.161	2.064
Use of toilet	1.40	1.20	0.626	1.752
Dressing lower body	1.18	1.27	0.794	1.844
Mobility in bed	1.02	1.16	0.602	1.740
Grooming	0.72	0.50	0.074	0.983
Bathing upper body	0.63	0.56	0.223	0.902
Bathing lower body	0.54	0.75	0.231	1.256
Injury severity	0.33	0.48	-0.091	1.120
Stair management	0.32	0.20	-0.525	0.891
Injury level	0.14	0.17	-0.244	0.686
Transfer ground-wheelchair	-0.36	-0.58	-1.147	0.023
Transfer wheelchair-car	-0.51	-0.52	-1.012	0.007
Age	-0.57	-0.67	-1.080	-0.251
Mobility indoors	-0.83	-0.90	-1.216	-0.484
Transfer wheelchair-toilet	-0.84	-0.87	-1.247	-0.214
Respiration	-0.85	-0.76	-1.176	-0.273
Bladder management	-1.01	-0.88	-1.315	-0.394
Bowel management	-1.01	-0.90	-1.279	-0.492
Mobility moderate distances	-1.01	-1.08	-1.314	-0.686
Mobility outdoors	-1.01	-1.07	-1.297	-0.736
Sex	-1.01	-0.92	-1.266	-0.290
Transfer bed-wheelchair	-1.01	-0.71	-1.172	-0.147

Abbreviation: CI, confidence interval; T1, Swiss Spinal Cord Injury Cohort Study assessment time point 1.

B) T4.

Node	Sample estimate	Bootstrap mean estimate	Bootstrap lower limit of 95% CI	Bootstrap upper limit of 95% CI
Feeding	1.63	1.51	1.028	1.940
Dressing lower body	1.53	1.45	0.941	1.948
Bathing lower body	1.42	1.35	0.892	1.835
Use of toilet	1.31	1.16	0.617	1.677
Dressing upper body	1.10	1.09	0.470	1.698
Mobility in bed	0.91	0.92	0.408	1.423
Stair management	0.81	0.71	0.224	1.253
Injury severity	0.68	0.87	0.338	1.530
Grooming	0.38	0.48	0.042	0.896
Bathing upper body	0.24	0.14	-0.337	0.588
Transfer ground-wheelchair	0.22	0.19	-0.309	0.627
Mobility outdoors	-0.08	-0.08	-0.555	0.381
Injury level	-0.35	-0.31	-0.849	0.361
Bladder management	-0.78	-0.54	-1.047	-0.002
Transfer bed-wheelchair	-0.83	-0.88	-1.354	-0.378
Age	-0.86	-1.28	-1.769	-0.771
Bowel management	-0.92	-0.78	-1.200	-0.241
Transfer wheelchair-car	-1.00	-0.79	-1.288	-0.160
Mobility indoors	-1.08	-1.07	-1.313	-0.476
Mobility moderate distances	-1.08	-1.14	-1.326	-0.832
Respiration	-1.08	-1.01	-1.258	-0.626
Sex	-1.08	-1.07	-1.334	-0.590
Transfer wheelchair-toilet	-1.08	-0.90	-1.246	-0.434

Abbreviation: CI, confidence interval; T4, Swiss Spinal Cord Injury Cohort Study assessment time point 4.
