# Supplementary material for: Revisiting functioning recovery in persons with spinal cord injury undergoing first rehabilitation: Trajectory and network analysis of a Swiss cohort study
Source: PLoS One. 2024 Feb 9;19(2):e0297682. doi: 10.1371/journal.pone.0297682 (PMC10857630; doi:10.1371/journal.pone.0297682)
Supplement: S1 Appendix — A) Final trajectory analysis model based on R package lcmm version 2.0.0. B) Final network analysis model for the moderate functioning improvement class based on R package bootnet version 1.5. (PDF) [file pone.0297682.s011.pdf]

S3 Appendix. Summary of R Syntax for final trajectory and network models.

A) Final trajectory analysis model based on R package lcmm version 2.0.0.[1]

```
#-----------------------------------------------------------------------------------------------------------------------------------------------
#Packages
#-----------------------------------------------------------------------------------------------------------------------------------------------
#Loading lcmm package
library(lcmm)

#-----------------------------------------------------------------------------------------------------------------------------------------------
#Reading in the formatted data (long format)
#-----------------------------------------------------------------------------------------------------------------------------------------------
#Loading the dataset data_long with variables:
#SCIM_rasch = Interval-based Spinal Cord Independence Measure version III (SCIM III)
#days_SCIM_adm = Time of assessment of SCIM III observation in days since admission to initial rehabilitation
#id_swisci_num = Participant ID
#PATH stands for the path to the data folder[2]
load("PATH/Formatting_2021-C-006_2022_03_16.Rda")
data_long <- data_traject_analysis_long

#Check sample size
length(unique(data_long$id_swisci))
# [1] 1099

#-----------------------------------------------------------------------------------------------------------------------------------------------
#------------------------------------------------------------------------Latent Process Mixed Model-----------------------------------------------
#-----------------------------------------------------------------------------------------------------------------------------------------------
#Model with 1 class
#SCIM_rasch~days_SCIM_adm, fixed effects
#random=~days_SCIM_adm, random effects
#subject='id_swisci_num', participant ID
#ng =1, one latent class
#idiag = F, unstructured random effects variance-covariance matrix
#data=data_long, estimation based on data_long
#link= '2-quant-splines', specification of parameterized link function
m1spl2q<- lcmm(SCIM_rasch~days_SCIM_adm, random=~days_SCIM_adm,
          subject='id_swisci_num', ng=1, idiag = F, data=data_long, link='2-quant-splines')

#Model with 4 classes
# gridsearch function, model estimation based on random initial values generated based on model with 1 class (250 random initial values;
200 iterations)
#mixture=~days_SCIM_adm, mixture effects
#ng = 4, four latent classes
#nwg = T, class-specifc (proportional) random effects variance-covariance matrix
m4spl2q<-gridsearch(rep = 250, maxiter = 200, minit = m1spl2q,
            lcmm(SCIM_rasch~days_SCIM_adm, random=~days_SCIM_adm, mixture=~days_SCIM_adm,
              subject='id_swisci_num', ng=4, nwg = T, idiag = F, data=data_long, link='2-quant-splines'))
```

[1] Proust-Lima C, Philipps V, Liquet B. Estimation of Extended Mixed Models Using Latent Classes and Latent Processes: The R Package lcmm. J Stat Softw. 2017;78(2):1-56. doi: 10.18637/jss.v078.i02.
[2] Not shown in this appendix.

B) Final network analysis model for the moderate functioning improvement class based on R package bootnet version 1.5.[3]

```
#----------------------------------------------------------------------------------------------------------------------------------------
#Packages
#----------------------------------------------------------------------------------------------------------------------------------------
#Loading packages
library(bootnet)

#----------------------------------------------------------------------------------------------------------------------------------------
#Reading the imputed data for T1 and T4
#----------------------------------------------------------------------------------------------------------------------------------------
#Previosly imputed datasets contain for T1 (admission) and T4 (discharge):
#Participant ID
#Age at injury
#Sex
#Injury level at T1/T4
#Injury severity at T1/T4
#19 items of SCIM III at T1/T4
#Class membership variable

#Load dataset T1
#PATH stands for the path to the data folder[4]
load("PATH/Preparation_Network_Analysis_4class_T1.Rda")
data_analysis_T1 <- data_swisci_analysis_imputed

#Check numbers of participants per latent trajectory class at T1
table(data_analysis_T1$N)
# 1   2   3   4
# 33 225  67 721

#Load dataset T4
load("PATH/Preparation_Network_Analysis_4class_T4.Rda")
data_analysis_T4 <- data_swisci_analysis_imputed

#Check numbers of participants per latent trajectory class at T4
table(data_analysis_T4$N)
# 1   2   3   4
# 32 235  74 746

#----------------------------------------------------------------------------------------------------------------------------------------
#Setting: Variables of interest and selecting latent trajectory class of interest (moderate functioning improvement class)
#----------------------------------------------------------------------------------------------------------------------------------------
#List of variables of interest
var_id <- c("id_swisci")
var_info <- c("age_sci","sex")
var_injury_t1 <- c("t1_neurol_level", "t1_neurol_ais")
var_injury_t4 <- c("t4_neurol_level", "t4_neurol_ais")
var_scim_t1 <- c("t1_scim_feeding", "t1_scim_bathing_upper", "t1_scim_bathing_lower",
         "t1_scim_dressing_upper", "t1_scim_dressing_lower", "t1_scim_grooming",
         "t1_scim_respiration", "t1_scim_bladder", "t1_scim_bowel", "t1_scim_toilet",
         "t1_scim_mobility_bed", "t1_scim_bed_wheelchair", "t1_scim_wheelchair_toilet",
         "t1_scim_indoor", "t1_scim_moderate", "t1_scim_outdoor",
         "t1_scim_stairs", "t1_scim_wheelchair_car", "t1_scim_ground_wheelchair")
var_scim_t4 <- c("t4_scim_feeding", "t4_scim_bathing_upper", "t4_scim_bathing_lower",
           "t4_scim_dressing_upper", "t4_scim_dressing_lower", "t4_scim_grooming",
           "t4_scim_respiration", "t4_scim_bladder", "t4_scim_bowel", "t4_scim_toilet",
           "t4_scim_mobility_bed", "t4_scim_bed_wheelchair", "t4_scim_wheelchair_toilet",
           "t4_scim_indoor", "t4_scim_moderate", "t4_scim_outdoor",
           "t4_scim_stairs", "t4_scim_wheelchair_car", "t4_scim_ground_wheelchair")

#Selecting moderate functioning improvement class (N=4):
#T1
data_analysis_t1_class4 <- subset(data_analysis_T1, N == 4)
dim(data_analysis_t1_class4)
# [1] 721  25

#T4
data_analysis_t4_class4 <- subset(data_analysis_T4, N == 4)
dim(data_analysis_t4_class4)
# [1] 746  25
```

[3] Epskamp S, Borsboom D, Fried EI. Estimating psychological networks and their accuracy: A tutorial paper. Behav Res Methods. 2018;50(1):195-212. doi: 10.3758/s13428-017-0862-1.
[4] Not shown in this appendix.

```
#Selecting network analysis variables:
#T1
data_analysis_t1_class4_functioning <-
        data_analysis_t1_class4[,which(names(data_analysis_t1_class4)%in%c(var_info,var_injury_t1,var_scim_t1))]
#T4
data_analysis_t4_class4_functioning <-
        data_analysis_t4_class4[,which(names(data_analysis_t4_class4)%in%c(var_info,var_injury_t4,var_scim_t4))]

#----------------------------------------------------------------------------------------------------------------------------------------------------
#Network Analysis - 4-Class Model, Class 4 - T1 - Tuning = 0
#----------------------------------------------------------------------------------------------------------------------------------------------------
#Transform data to numeric format
data_analysis_t1_class4_functioning[,c(var_info,var_injury_t1,var_scim_t1)] <-
        sapply(sapply(sapply(data_analysis_t1_class4_functioning[,c(var_info,var_injury_t1,var_scim_t1)],as.character), unclass),
        as.numeric)

network1_t1_class4 <- estimateNetwork(data_analysis_t1_class4_functioning,
                default = "mgm", criterion = "EBIC",
                binarySign = TRUE,
                level = c(1,2,rep(1,21)),
                type = c("g","c",rep("g",21)),
                tuning = 0,
                transform = "rank")

#----------------------------------------------------------------------------------------------------------------------------------------------------
#Network Analysis - 4-Class Model, Class 4 - T4 - Tuning = 0
#----------------------------------------------------------------------------------------------------------------------------------------------------
#Transform data to numeric format
data_analysis_t4_class4_functioning[,c(var_info,var_injury_t4,var_scim_t4)] <-
        sapply(sapply(sapply(data_analysis_t4_class4_functioning[,c(var_info,var_injury_t4,var_scim_t4)],as.character), unclass),
        as.numeric)

network1_t4_class4 <- estimateNetwork(data_analysis_t4_class4_functioning,
                default = "mgm", criterion = "EBIC",
                binarySign = TRUE,
                level = c(1,2,rep(1,21)),
                type = c("g","c",rep("g",21)),
                tuning = 0,
                transform = "rank")
```
