# Supplementary material for: Revisiting functioning recovery in persons with spinal cord injury undergoing first rehabilitation: Trajectory and network analysis of a Swiss cohort study
Source: PLoS One. 2024 Feb 9;19(2):e0297682. doi: 10.1371/journal.pone.0297682 (PMC10857630; doi:10.1371/journal.pone.0297682)
Supplement: S1 Fig — Abbreviations: SCIM III, Spinal Cord Independence Measure version III. (PDF) [file pone.0297682.s012.pdf]

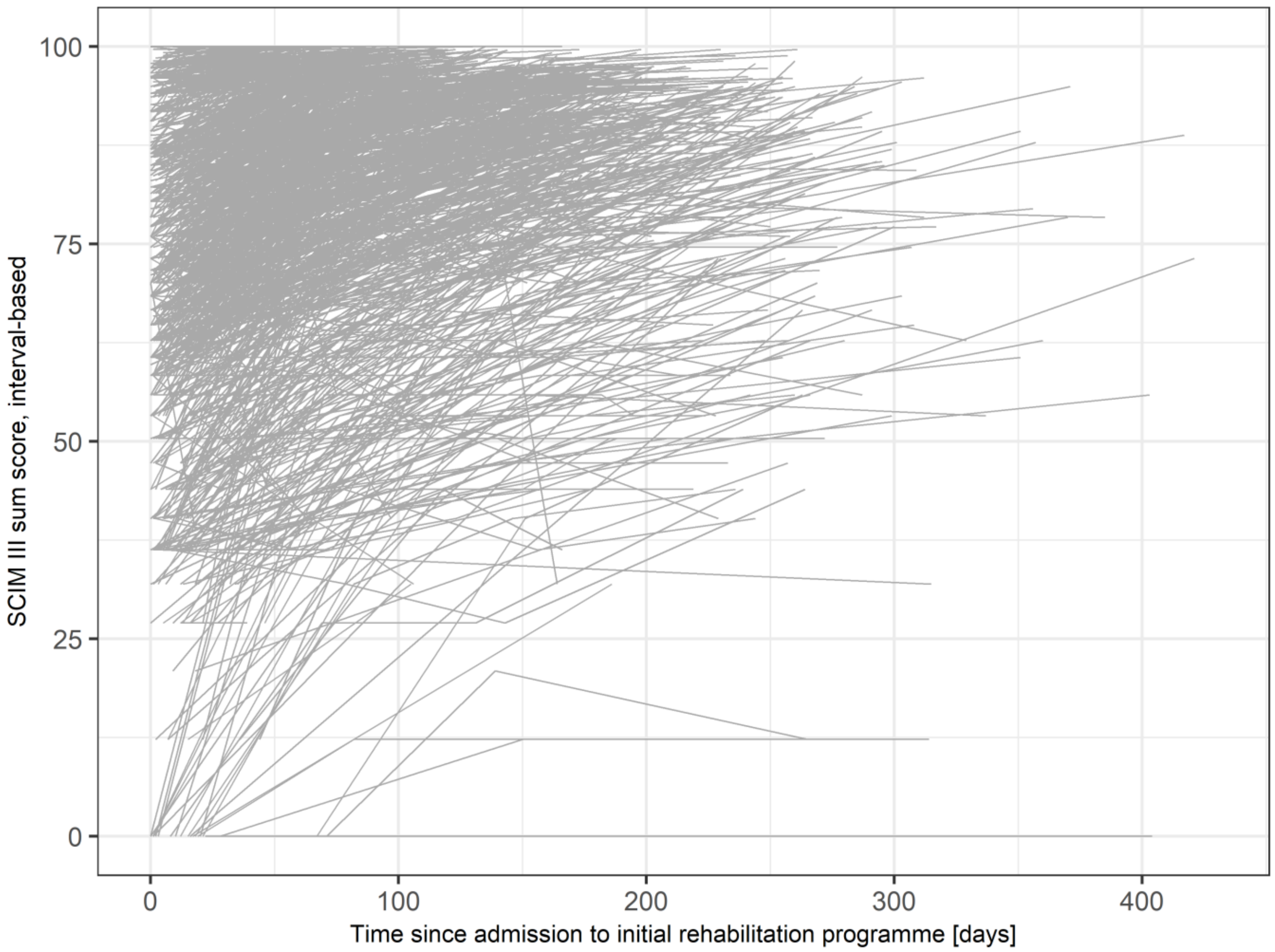

S6 Fig. Observed individual functioning trajectories according to Rasch-transformed SICM III total scores (N=1099). Abbreviations: SCIM III, Spinal Cord Independence Measure version III.
