# Supplementary material for: Revisiting functioning recovery in persons with spinal cord injury undergoing first rehabilitation: Trajectory and network analysis of a Swiss cohort study
Source: PLoS One. 2024 Feb 9;19(2):e0297682. doi: 10.1371/journal.pone.0297682 (PMC10857630; doi:10.1371/journal.pone.0297682)
Supplement: S2 Fig — Abbreviations: AIC, Akaike information criterion; SCIM III, Spinal Cord Independence Measure version III. (PDF) [file pone.0297682.s013.pdf]

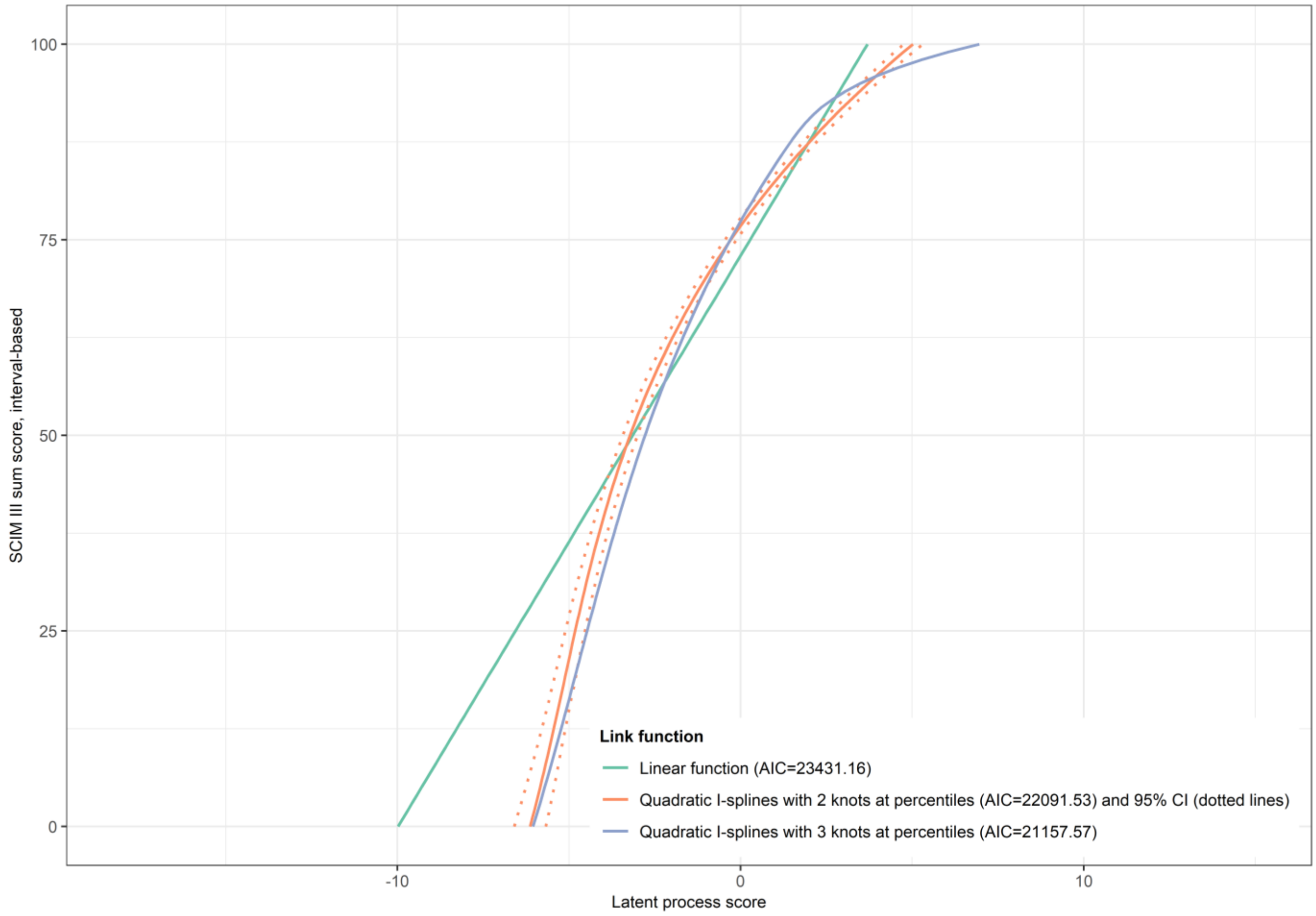

S7 Fig. Estimated parameterized link functions. Abbreviations: AIC, Akaike information criterion; SCIM III, Spinal Cord Independence Measure version III.
