# Supplementary material for: Revisiting functioning recovery in persons with spinal cord injury undergoing first rehabilitation: Trajectory and network analysis of a Swiss cohort study
Source: PLoS One. 2024 Feb 9;19(2):e0297682. doi: 10.1371/journal.pone.0297682 (PMC10857630; doi:10.1371/journal.pone.0297682)
Supplement: S4 Fig — A) one class. B) two classes. C) three classes. D) four classes. E) five classes. F) six classes. Abbreviations: SCIM III, Spinal Cord Independence Measure version III. (PDF) [file pone.0297682.s015.pdf]

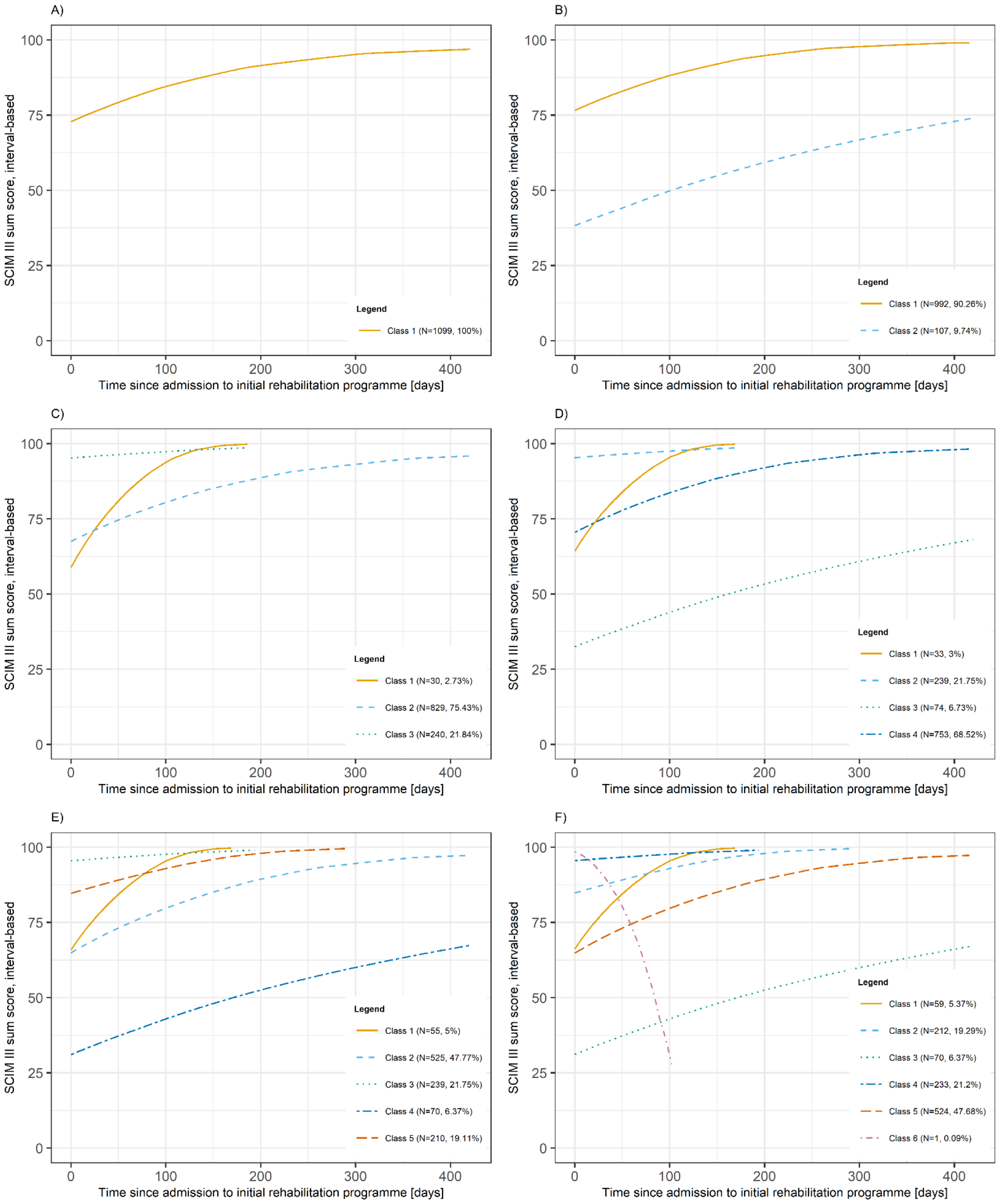

S9 Fig. Predicted mean functioning trajectories of latent process mixed models with varying between-person trajectory variability across classes (set 2). A) one class. B) two classes. C) three classes. D) four classes. E) five classes. F) six classes. Abbreviations: SCIM III, Spinal Cord Independence Measure version III.
