# Supplementary material for: Revisiting functioning recovery in persons with spinal cord injury undergoing first rehabilitation: Trajectory and network analysis of a Swiss cohort study
Source: PLoS One. 2024 Feb 9;19(2):e0297682. doi: 10.1371/journal.pone.0297682 (PMC10857630; doi:10.1371/journal.pone.0297682)
Supplement: S5 Fig — A) T1. B) T4. The lines on the y-axis indicate the different edges (names not shown) in the respective network and the x-axis indicated the corresponding edge weights. The red line shows the estimated edge weights based on the original sample of each network, the black dots and grey bars show the bootstrap mean edge weights and corresponding 95% bootstrap confidence intervals, respectively. Abbreviations: SwiSCI Swiss Spinal Cord Injury Cohort Study; T1, T4, SwiSCI assessment time points 1, 4. (PDF) [file pone.0297682.s016.pdf]

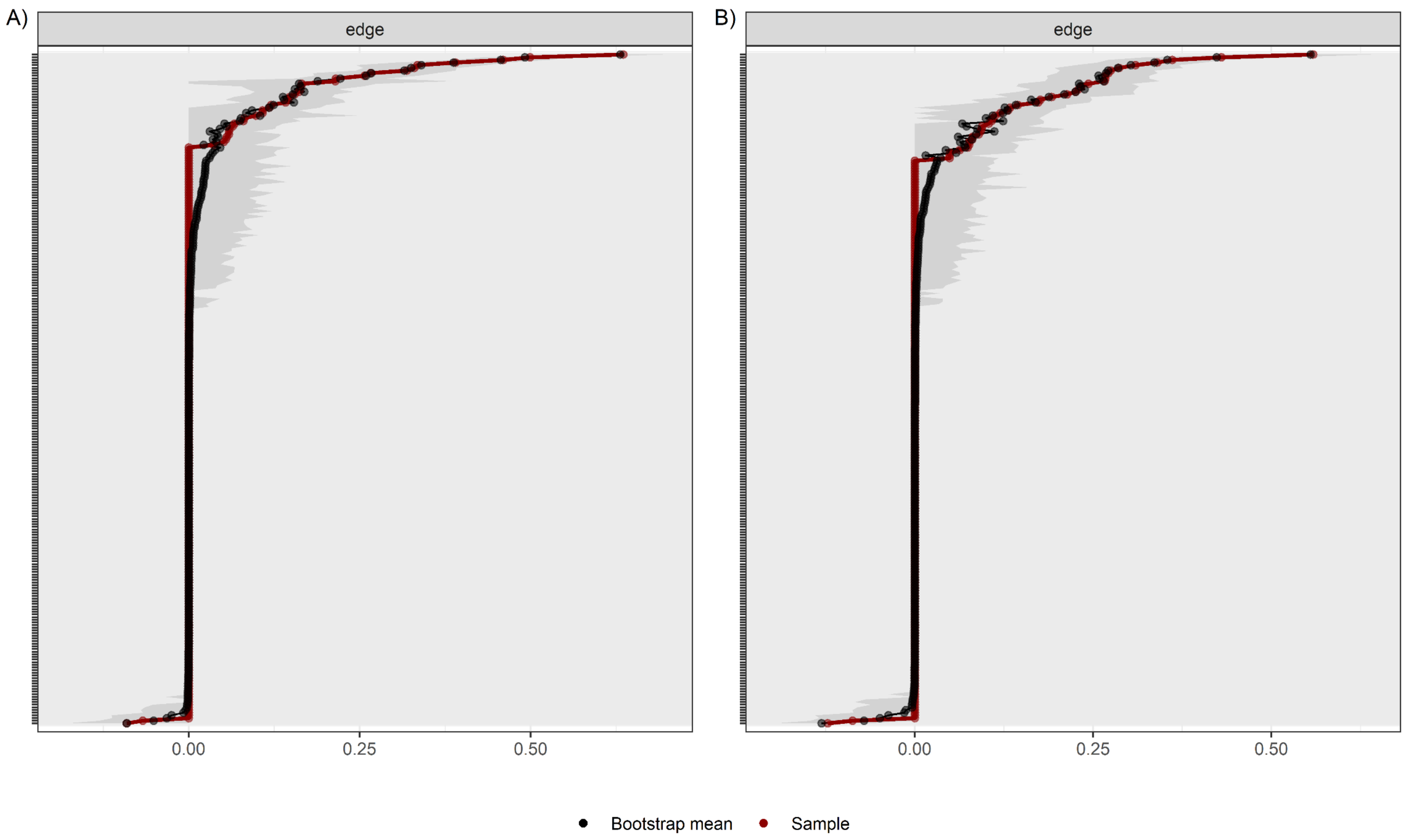

S14 Fig. Estimated edge weight accuracy based on the mixed graphical model networks for the moderate functioning improvement class. A) T1. B) T4. The lines on the y-axis indicate the different edges (names not shown) in the respective network and the x-axis indicated the corresponding edge weights. The red line shows the estimated edge weights based on the original sample of each network, the black dots and grey bars show the bootstrap mean edge weights and corresponding 95% bootstrap confidence intervals, respectively. Abbreviations: SwiSCI Swiss Spinal Cord Injury Cohort Study; T1, T4, SwiSCI assessment time points 1, 4.
