# Supplementary material for: Revisiting functioning recovery in persons with spinal cord injury undergoing first rehabilitation: Trajectory and network analysis of a Swiss cohort study
Source: PLoS One. 2024 Feb 9;19(2):e0297682. doi: 10.1371/journal.pone.0297682 (PMC10857630; doi:10.1371/journal.pone.0297682)
Supplement: S6 Fig — A) T1. B) T4. Abbreviations: SwiSCI Swiss Spinal Cord Injury Cohort Study; T1, T4, SwiSCI assessment time points 1, 4. (PDF) [file pone.0297682.s017.pdf]

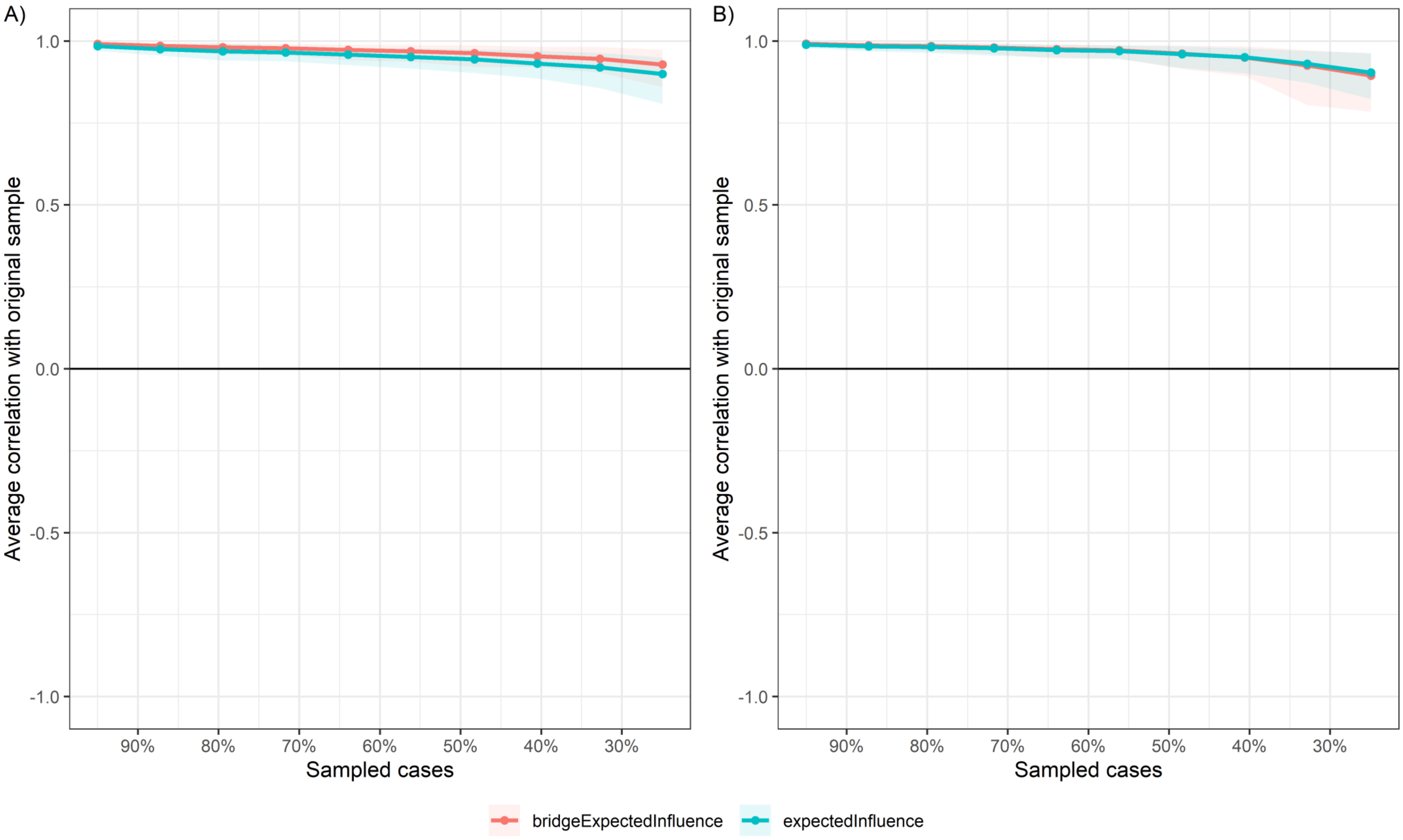

S17 Fig. Bootstrapped stability of centrality indices based on the mixed graphical model networks for the moderate functioning improvement class. A) T1. B) T4. Abbreviations: SwiSCI Swiss Spinal Cord Injury Cohort Study; T1, T4, SwiSCI assessment time points 1, 4.
