# Supplementary material for: Revisiting functioning recovery in persons with spinal cord injury undergoing first rehabilitation: Trajectory and network analysis of a Swiss cohort study
Source: PLoS One. 2024 Feb 9;19(2):e0297682. doi: 10.1371/journal.pone.0297682 (PMC10857630; doi:10.1371/journal.pone.0297682)
Supplement: Attachment — Submitted filename: Response to Reviewers.pdf [file pone.0297682.s018.pdf]

Revisiting functioning recovery in persons with spinal cord injury undergoing first rehabilitation: Trajectory and network analysis of a Swiss cohort study

Reply to the reviewers' comments:
The line numbers within the authors' reply refer to the revised manuscript with tracked and highlighted changes. At the end of the document, please find in addition our response to the comments about the journal requirements.

Reviewer 1	Authors' reply
**Remark 1:**<br>The title is OK but should be revised to highlight the association that is being studied between functional trajectories and the functioning domains. The following words "a network analysis approach" are not as important as the primary finding of this study. Especially, the network analysis is only performed on two groups of the four.	Thank you for your comment and the suggestion to revise the manuscript title (*Revisiting functioning trajectories in persons with spinal cord injury undergoing first rehabilitation in Switzerland: A network analysis approach*). We agree that the title does not reflect all parts of the analysis equally. However, we chose the phrase "A network analysis approach" to emphasize what is new about the current analysis, compared to what has been done previously. Therefore, we propose the following revised manuscript title: *Revisiting functioning recovery in persons with spinal cord injury undergoing first rehabilitation: Trajectory and network analysis of a Swiss cohort study.*
**Remark 2: Overall impressions**<br>The study aimed to evaluate functioning trajectories of individuals with SCI undergoing rehabilitation. The emphasis seems to be on categorizing individuals into one of four groups (high functioning, early, etc.). This study confirmed prior findings with updated datasets. While these findings are important, their contribution to improving clinical care is limited due to the lack of factors such as level and completeness of injury that were not considered in the functional recovery trajectory analyses. The study also identified functioning domains that affect the trajectory analysis. However, it is unclear if these functioning domains can be improved without considering the other SCI-related and patient-related factors towards functional improvement in these domains.	This is a very important comment. We agree that in order to assess the clinical relevance of the identified functioning trajectories, it is essential to make the link to patient-related as well as injury-specific covariates. Therefore, in the trajectory analysis we have included a table (Table 3) that shows the patient and injury related characteristics together with other relevant factors for each of the identified functioning trajectory classes. Typically, predictors of class membership are examined in a separate and additional analysis, such as multinomial logistic regression. Due to the very limited sample size of one of our identified functioning trajectory classes (N=33 for the *early functioning improvement class*), we refrained from such an analysis. Instead, we mention important trajectory class differences for further investigations in the Discussion section. *(Lines 504-519)*<br><br>The network analysis of the *stable high* and the *moderate improvement classes* included patient characteristics (age, sex) as well as injury characteristics (injury level and severity). Based on your comment, we have decided to specifically highlight these variables in the revised version of the manuscript by adding the following statement in the Methods section: *In addition, patient- (sex, age) as well as injury-related (injury level and severity) covariates were included in all network models. (Lines 157-158)*
**Remark 3: Introduction**<br>This section is well written and highlights the article's focus on tracking functional recovery and identifying functional domains related to recovery. While identifying functional domains associated with recovery is important, the article misses the opportunity to study	We agree that the missing link between identified functioning trajectories and patient- and SCI-related factors is a limitation of our work. We have added a corresponding note in the Limitations section: *In addition, we did not investigate potential predictors for*

SCI-related and patient-related characteristics that are associated with functional recovery.<br>Clinicians might not be able to predict if their treatment will yield better outcomes without the knowledge of how the functional recovery is affected by SCI-related (e.g. level and completeness) and patient-related characteristics (e.g. age).	*class membership due to the small sample sizes of identified functioning trajectories. This further limits the interpretability of functioning trajectories regarding important patient- and injury-related characteristics, such as age or injury severity, and their applicability in practice and corresponding studies are needed to clarify these questions. (Lines 640-644)*
**Remark 4: Methods**<br>The section clearly describes the time points and measures used in the study.<br>Rasch and Trajectory analyses are described clearly. Did the distribution of the functional recovery (within the dataset available to the researchers) affect the calibration.	 Thank you for your question. In order to prevent the distribution of functioning recovery from influencing the Rasch analysis, the calibration sample (N=400) was specifically designed so that the SCIM III scores were evenly distributed in the sample. The same was done for the time points T1, T2, T3, T4. This was done to ensure that the calibration sample covered the full range of SCIM III scores and all available time points. We have slightly reformulated the corresponding part of the manuscript to enhance transparency: *The calibration sample for each analysis included N=400 observations of available participants from the SwiSCI Inception Cohort or the MDS assessment and was developed based on random sampling with equal distributions of assessment time points (T1-T4) and SCIM III scores without extremes (1-99). (Lines 165-169)*<br><br>Regarding the trajectory analysis, we took care of the bounded, skewed distribution of SCIM III by using a parametrized link function in the formulation of the latent class mixed model.
**Remark 5: Results**<br>The Rasch analysis indicates the robustness of the SCIM III total scores for this population.<br>The trajectory analysis indicated the four-class model is preferred. Did the small sample size for early functioning and slow functioning affect the classification analysis. Would it have been better to do a two-class model for high functioning (including early function) and moderate to slow functioning.<br>Figure 3 does some analysis with sex and other variables such as injury severity. An analysis that includes both the injury severity (AIS) level and the neurological level of injury should be evaluated.	 This is an interesting consideration. The small sample sizes of the *early* and *slow recovery classes* were the result of the trajectory analysis and therefore they did not necessarily affect the classification analysis as such. Also, the final model shows good classification accuracy with an entropy of 0.8 and, as shown in Table S9, with mean posterior class membership probabilities between 78.91% (*early functioning improvement class*) and 91.30% (*moderate functioning improvement class*), despite the relatively small sample for two of the identified classes.<br>Generally, in the trajectory analysis several models are fit, each with a different assumption about the number of latent classes in the study sample. The final number of classes is decided based on the best model fit. In our sample, the two-class model showed a worse fit than the four-class model in almost all indices, such as BIC, SSABIC, and AIC (see Table 2), and only the entropy value was higher in this model. Furthermore, also in the two-class model, one of the identified classes was rather small compared to the other class (N=107, 9.74% vs. N=992, 90.26%). For all these reasons, we decided to use the four-class model as the final model. In conclusion, a two-class model may be easier to understand and to perform subsequent analyses, but it may be too simplistic and would not show the optimal fit to the observations at hand.

	Regarding the inclusion of patient- and injury-related covariates in the network analysis, as mentioned in our response to Remark 2, we have added a clarifying statement in the Methods section. *(Lines 157-158)*
**Remark 6: Discussion**<br>Highlights their findings and the differences that were observed compared to previous research.<br>This section does a good job of comparing their findings with other research such as Tulsky et al. and the development of SCIM III.<br>The network analysis is interesting but is limited to just the two groups, which should be highlighted in this section as well.<br>As indicated in the limitation section a network analysis might connect sub-scores of SCIM III (domains) to the functional recovery trajectories but do not reflect the impact of specific rehabilitation interventions. This should be discussed in the article.<br>The section should highlight that a more detailed study is necessary to study the impact of other factors on functional recovery.	Thank you for your comment and the suggestions for improving the Discussion and Limitations sections of the manuscript. Based on your comment, we have made the following changes and additions:<br>• In the Discussion section about the network analysis, we now emphasized the fact that the analysis was performed for only two groups and that future studies could address this limitation: *Future studies are needed to confirm our results, to extend the analysis to the early and slow functioning improvement classes and to investigate relevant functioning problems that could not be taken into account in our network analysis approach. (Lines 612-614)*<br>• As suggested, the explanation of the interpretation of the centrality indices has been clarified and moved to the Discussion section about the network analysis: *Centrality indices should be understood as a tool to summarize the association of a variable in a network according to the strength of its connections (edges). In this sense, they can point to interesting areas of functioning as targets for interventions, but they do not reflect interventions and their effects as such. Since edges in a network do not necessarily reflect causal relationships but may also indicate bidirectional or conditional relationships between nodes, or the presence of unmodelled latent variables [33], centrality indices in turn do not necessarily assess the impact caused by specific rehabilitation interventions in clinical practice. (Lines 590-597)*<br>• As mentioned in our response to Remark 3, we have added some discussion of the inclusion of other factors related to functioning recovery in the Limitations section. *(Lines 640-644)*<br><br>In addition, we used the reviewers' feedback as an opportunity to critically review the Limitations section of our manuscript and we decided to add some clarifications and specifications on points that we felt were key to understanding the methods used.
**Remark 7: Conclusion**<br>This section should indicate that while the prior results are confirmed their use in the clinical care is limited without considering the SCI-related and patient-related factors.	We agree that the conclusion should also reflect some of the limitations of the study. Therefore, we have restructured the Conclusion paragraph and added the following statement: *In a next step it will be important to study the role that patient- and injury-related characteristics play in this understanding. (Lines 688-689)*

**Remark 8: References**<br>While the article does a good job of citing various references. It misses the opportunity to highlight some key research that is associated with functional recovery in individuals with SCI in the last few years.<br>• Early predictors of functional outcome after trauma. PM&R 2016;8(4):314–20.<br>• Linking individual data from the spinal cord injury model systems center and local trauma registry: development and validation of probabilistic matching algorithm. Top Spinal Cord Inj Rehabil 2020;26(4): 221–31.<br>• Evaluating associations between trauma-related characteristics and functional recovery in individuals with spinal cord injury. The Journal of Spinal Cord Medicine 2022.	Thank you for pointing us to these interesting publications. We decided to add one of them to the Limitations section to emphasize the point of including trauma and acute phase-related variables in future longitudinal analyses of functioning in persons with SCI, next to patient- and injury-related factors: *Moreover, such studies might in addition investigate the relationship between trauma and acute care variables with functioning trajectory classes, as they have been shown to be associated with functioning recovery [58]. (Lines 644-647)*
**Reviewer 2**	**Authors' reply**
**Remark 1:**<br>A very extended documentation of functioning trajectories for SCI patients. Although, it remains in the era of one country and a specific Healthcare System, is quite interesting.<br>The next step could be a comparative multi-country research to obtain a global aspect.	Thank you very much for your feedback and for sharing your idea for a follow-up project. Yes, we agree that a multi-country perspective could be interesting and relevant. Similar to the International Spinal Cord Injury Community Survey (InSCI) initiative, such a comparison could involve countries from all WHO regions.
**Journal requirements**	**Authors' reply**
**1.** Please ensure that your manuscript meets PLOS ONE's style requirements, including those for file naming.	Thanks for the information and the template files. We have checked and adapted our manuscript to meet the PLOS ONE style requirements.
**2.** Please note that PLOS ONE has specific guidelines on code sharing for submissions in which author-generated code underpins the findings in the manuscript. In these cases, all author-generated code must be made available without restrictions upon publication of the work. Please review our guidelines at https://journals.plos.org/plosone/s/materials-and-software-sharing#loc-sharing-code and ensure that your code is shared in a way that follows best practice and facilitates reproducibility and reuse.	This is a very important topic. In order to make our analyses transparent, we have added a document in the supplemental material of our manuscript that includes our R code used for the final trajectory and the network models (see S3 Appendix).<br>In addition, we have added the Supplemental Tables S13, S15, and S16, which show the detailed estimates used for Fig 3, 4, and 5.
**3.** Note from Emily Chenette, Editor in Chief of PLOS ONE, and Iain Hrynaszkiewicz, Director of Open Research Solutions at PLOS: Did you know that depositing data in a repository is associated with up to a 25% citation advantage (https://doi.org/10.1371/journal.pone.0230416)? If you've not already done so, consider depositing your raw data in a repository to ensure your work is read, appreciated and cited by the largest possible audience. You'll also earn an Accessible Data icon on your published paper if you deposit your data in any participating repository (https://plos.org/open-science/open-data/#accessible-data).	Thank you for the information about data sharing. Due to the commitment of the Swiss Spinal Cord Injury Cohort Study (SwiSCI) to protect study participants' privacy, we are not able to make our datasets publicly available.
**4.** In your Data Availability statement, you have not specified where the minimal data set underlying the results described in your manuscript can be found. PLOS	As described above, the dataset analyzed in this study are not publicly available due to the commitment of SwiSCI to protect the privacy of study participants but

defines a study's minimal data set as the underlying data used to reach the conclusions drawn in the manuscript and any additional data required to replicate the reported study findings in their entirety. All PLOS journals require that the minimal data set be made fully available. For more information about our data policy, please see http://journals.plos.org/plosone/s/data-availability.<br><br>Upon re-submitting your revised manuscript, please upload your study's minimal underlying data set as either Supporting Information files or to a stable, public repository and include the relevant URLs, DOIs, or accession numbers within your revised cover letter. For a list of acceptable repositories, please see http://journals.plos.org/plosone/s/data-availability#loc-recommended-repositories. Any potentially identifying patient information must be fully anonymized.<br><br>Important: If there are ethical or legal restrictions to sharing your data publicly, please explain these restrictions in detail. Please see our guidelines for more information on what we consider unacceptable restrictions to publicly sharing data: http://journals.plos.org/plosone/s/data-availability#loc-unacceptable-data-access-restrictions. Note that it is not acceptable for the authors to be the sole named individuals responsible for ensuring data access.<br><br>We will update your Data Availability statement to reflect the information you provide in your cover letter.	are available from the SwiSCI Study Center (contact@swisci.ch) on reasonable request. We thank you very much for your understanding in this regard.
**5.** One of the noted authors is a group [SwiSCI Study Group]. In addition to naming the author group, please list the individual authors and affiliations within this group in the acknowledgments section of your manuscript. Please also indicate clearly a lead author for this group along with a contact email address.	Thank you for this clarification. In the past, the inclusion of the SwiSCI Study Group in the author list was suggested by the SwiSCI Guidelines. However, we have been informed that these guidelines are currently being under revision and the addition of the statement is no longer suggested. Therefore, in consultation with the SwiSCI Study Center, it has been decided to remove the SwiSCI Study Group from the author list. Thank you for your understanding.
